# *RAD21L1* Is Sufficient and Effective for Reprogramming Human Sertoli Cells to Phenotypic Spermatogonial Stem Cells Through DNA Methylation and Essential for Male Fertility

**DOI:** 10.1002/advs.202417491

**Published:** 2025-10-16

**Authors:** Caimei He, Yinghong Cui, Wei Chen, Chunyun Li, Zuping He

**Affiliations:** ^1^ Hainan Academy of Sciences, School of Life Sciences and Medical Technology Hainan Medical University 352 Yehai Road, Longhua Distr Haikou 571199 China; ^2^ Key Laboratory of Model Animals and Stem Cell Biology in Hunan Province Engineering Research Center of Reproduction and Translational Medicine of Hunan Province Hunan Normal University School of Medicine Changsha 410013 China; ^3^ The Affiliated Hosptial of Hunan University Xiangtan Central Hospital Xiangtan 411100 China

**Keywords:** DNMT1, human Sertoli cell reprogramming, *RAD21L1*, *RAD21L1* mutations and male infertility, spermatogonial stem cells

## Abstract

It remains unknown about molecular mechanisms underlying the transition of somatic cells into male germ cells. It is observed that *RAD21L1* transcript is upregulated during reprogramming of Sertoli cells into human spermatogonial stem cells (SSCs) by overexpressing *DAZ* family genes. Significantly, *RAD21L1* overexpression transits Sertoli cells into phenotypic and functional human SSCs with high safety. RNA sequencing shows that *DNMT1* is expressed at a higher level by *RAD21L1* overexpression when Sertoli cells are reprogrammed to become human SSCs. Whole genome bisulfite sequencing elucidates that *RAD21L1* modulates DNA methylation to reprogram Sertoli cells into human SSCs, and *RAD21L1* interacts with DNMT1 in human SSCs generated from Sertoli cells. Intriguingly, *RAD21L1* mutation results in the decreases in stemness maintenance of human SSCs and DNMT1 expression levels. Notably, *RAD21L1* mutations are positively related to risk of non‐obstructive azoospermia (NOA) and male infertility. Collectively, these results implicate that *RAD21L1* is sufficient and effective for reprogramming human Sertoli cells to SSCs through modulating DNMT1 and *RAD21L1* mutations leads to NOA. This study is of particular significance because it provides a novel molecular mechanism that reprograms human somatic cells into human SSCs and it could offer invaluable gametes for treating male infertility.

## Graphical abstract

*RAD21L1* is upregulated in human Sertoli cells to be transited to become spermatogonial stem cells by overexpressing *DAZ* family three genes. *RAD21L1* is sufficient and effective for reprogramming Sertoli cells into human spermatogonial stem cells with high safety through DNA methylation. *RAD21L1* mutations have been identified to be associated with the risk of spermatogenesis disorders.

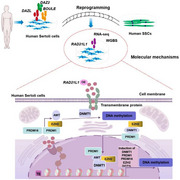

## 1 Introduction

Male Infertility has become a serious issue for human reproduction because around 50% of infertility might be resulted from male factors.^[^ ^1^ ^]^ Spermatogenesis comprises three main stages, namely, mitosis of SSCs, meiosis of spermatocytes, and spermiogenesis of round spermatids.^[^ ^2^ ^]^ Male infertility includes non‐obstructive azoospermia (NOA) with spermatogenesis failure and obstructive azoospermia (OA). Notably, Sertoli cell‐only syndrome (SCOS) has been regarded as the most prevalent and severe form of NOA. SCOS patients have Sertoli cells only within seminiferous tubules of human testes without any male germ cells.^[^ ^3^ ^]^ Currently, NOA patients without spermatids are unable to generate offspring with their own genetics. It has been demonstrated that pluripotent stem cells can be differentiated into primordial germ cell‐like cells (PGCLCs) to give rise to functional spermatids.^[^ ^4^ ^]^ The overexpression of the deleted in azoospermia (*DAZ*) family genes has been reported to facilitate the development of haploid gametes from pluripotent stem cells.^[^ ^5^ ^]^ Recently, we have demonstrated that overexpression of *DAZ* family three genes can reprogram Sertoli cells into human SSCs.^[^ ^6^ ^]^ It is worth noting that deletions or mutations of *DAZ* family genes cause spermatogenesis disorders, for example, severe azoospermia or oligospermia. *DAZ* family genes are composed of three members that is, *DAZ2*, Y chromosome‐linked *DAZ* and autosomal *BOULE*, which are involved in gene regulation during spermatogenesis.^[^ ^7^ ^]^ DAZ2 (formerly named SPGY1) is related to the cross‐reacting tail protein, and it is located on sperm tails.^[^ ^8^ ^]^ It is important to uncover the molecular mechanisms that reprogram human somatic cells to SSCs and other male germ cells, which can close the gap between male germ cells and somatic cells and offer a novel strategy to acquire phenotypic and functional spermatozoa for patients of azoospermia.

Reprogramming can change the cellular identities of cells to produce specific and functional cells. Novel methodologies have been developed for cell reprogramming, including the overexpression of specific transcription factors, the applications of small chemical molecules, and gene editing techniques. Human fibroblasts can be reprogrammed to become the induced pluripotent stem cells (iPS cells) through nuclear transfer and overexpression of transcription factors,^[^ ^9^ ^]^ which is a milestone for somatic cells to be utilized in regenerative medicine. Reprogramming techniques are pivotal in addressing male infertility, which can offer important and new solutions for these patients. The three‐step differentiation strategy has been devised to effectively induce human iPS cells to differentiate into functional spermatids.^[^ ^10^ ^]^ Overexpression of *DAZ* family genes can induce human embryonic stem (ES) cells to differentiate into haploid gametes.^[^ ^5^ ^]^ Moreover, overexpressing *DAZ* family genes can reprogram mesenchymal stem cells (MSCs) to produce germ cells.^[^ ^11^ ^]^ Small chemical molecules have been employed to transit somatic cells into the iPS cells that have significant applications in regenerative medicine.^[^ ^11^ ^]^ Human somatic cells can be reprogrammed by JNK inhibitor to become pluripotent stem cells.^[^ ^12^ ^]^ Nevertheless, it remains unknown about molecular mechanisms underlying the transition of human somatic cells into human male germ cells. Here we are the first to report that *RAD21L1* is essential for directly reprogramming Sertoli cells to human SSCs through DNA methylation.

*RAD21L1* is mainly expressed in mammalian testicular tissues. Interestingly, *RAD21L1* is RAD21 Papal homologs, and the BLAST sequence alignment shows that *RAD21L1* is similar to RAD21. Here we found that *RAD21L1* transcription was enhanced when Sertoli cells were reprogrammed into human SSCs by overexpressing *DAZ* family genes. More significantly, we have demonstrated that overexpression of only *RAD21L1* gene was sufficient to directly transit Sertoli cells into phenotypic and functional human SSCs with high safety. Our RNA sequencing highlighted that genes for pluripotency and stem cells, for example, *OCT4* (*POU5F1*), *LIN28A*, *WNT9*, and *FGF2*, as well as *ESRRB*, a biomarker of reprogramming, were expressed at higher levels by *RAD21L1* overexpressing. Furthermore, the transcripts of DNA and histone methylation or methytransferase activity related genes, including *DNMT1*, *PRDM16*, *PRDM1*, *EZH2*, *DOT1L*, and *AMT*, were significantly increased by *RAD21L1* overexpressing. Our WGBS implicates that *RAD21L1* reprogrammed human Sertoli cells into SSCs via DNA methylation. DNA methyltransferases (DMNTs) catalyze DNA methylation that is involved in spermatogenesis and male fertility. Five different DNMTs have been identified in mammals, with *DNMT1* primarily responsible for maintaining methylation.^[^ ^13^ ^]^ Interestingly, *DNMT1* transcripts are enriched in male germ cells.^[^ ^14^ ^]^ Epigenetic regulation of the genome is crucial for somatic cell reprogramming.^[^ ^15^ ^]^ In this study, we are the first to show that *DNMT1* is a key factor involved in the *RAD21L1*‐mediated DNA methylation, which reprograms Sertoli cells into human SSCs. Significantly, we found that *RAD21L1* mutations were closely associated with the risk of NOA and male infertility. As such, this study offers a novel genetic and epigenetic mechanism that mediates the reprogramming of human somatic cells into human germline stem cell, and significantly, it might provide invaluable male gametes for NOA patients to restore male fertility.

## 2 Results

### 2.1 *RAD21L1* Transcript Is Upregulated by Overexpressing *DAZ* Family Genes during Reprogramming of Sertoli Cells into Human SSCs

We have reported that primary human Sertoli cells could be reprogrammed into human SSCs phenotypically in vivo and in vitro via overexpressing *DAZ* family three genes, namely, *DAZ*, *DAZ2* and *BOULE*.^[^ ^6^ ^]^ However, it remains unknown about molecular mechanisms by which *DAZ* family genes transit Sertoli cells to human SSCs.

**Figure 1:**
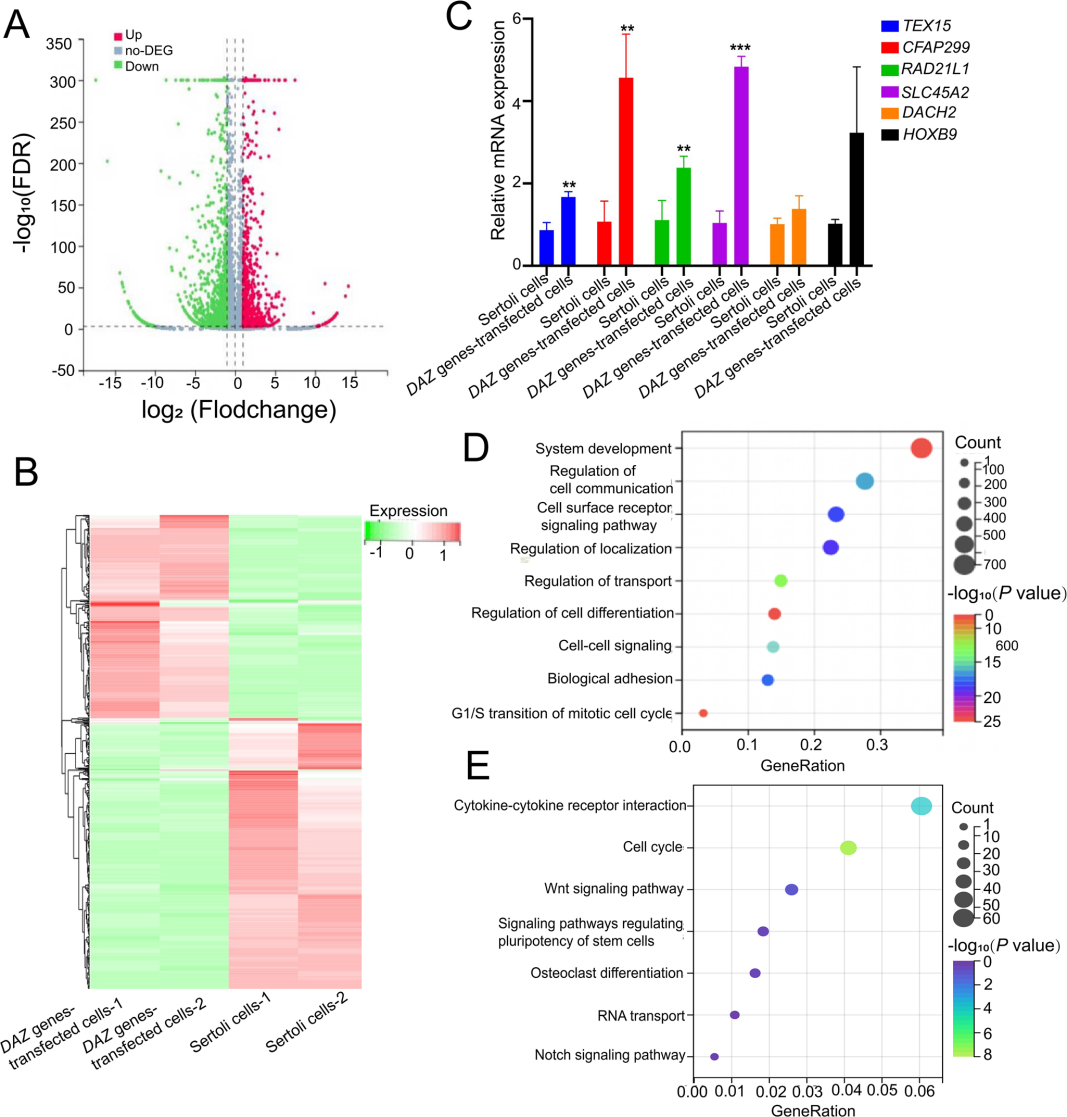
*DAZ* genes‐transfected cells have different transcriptomes compared to human Sertoli cells and *RAD21L1* is upregulated by overexpressing *DAZ* family genes. A) The volcano plot demonstrated the DEGs between *DAZ* genes‐transfected cells and human Sertoli cells. B) Hierarchical clustering showed the DEGs between *DAZ* genes‐transfected cells and human Sertoli cells. Notes: Red color represented a relatively higher expression levels of genes, whereas blue color indicated a relatively lower expression levels of genes. C) The qPCR revealed mRNA expression of *RAD21L1*, *TEX15*, *CFAP299*, *SLC45A2*, *DACH2*, and *HOXB9* in *DAZ* genes‐transfected cells and human Sertoli cells. D) KEGG pathway analysis of the DEGs between *DAZ* genes‐transfected cells and human Sertoli cells. The items with larger bubbles indicated more DEGs. The bubble colors were changed from purple, blue, green to red, which reflected the smaller the enrichment *P* value. E) GO analysis showed the top 7 enrichment functional terms.

We sought to identify key regulators for reprogramming human Sertoli cells into human SSCs. Our RNA‐seq revealed that there were 1972 differentially expressed genes (DEGs) between *DAZ* genes‐transfected cells and human Sertoli cells. Volcano plots (**Figure** **1**A) and heat map (Figure 1A,B) of the DEGs illustrated that 911 genes were upregulated and 1061 genes were downregulated in the *DAZ* genes‐transfected cells compared to human Sertoli cells. Several upregulated genes by *DAZ* family genes, as identified by RNA‐seq, including *RAD21L1* (Fold change: 8.856, *p* = 0.0089), *TEX15*, *CFAP299* (*c40rf22*), *SLC45A2*, *DACH2*, and *HOXB9*, were verified by qPCR (Figure 1C). KEGG and Gene Ontology (GO) analyses revealed that these DEGs were mainly enriched in signaling pathways regulating pluripotency of stem cells, cell communication, and cell differentiation (Figure 1D,E). Considered together, we demonstrated that *RAD21L1* gene is upregulated by overexpressing *DAZ* family genes when Sertoli cells were reprogrammed into human SSCs.

### 2.2 *RAD21L1* Is Expressed in Human SSCs and It Can Be Overexpressed in Human Sertoli Cells

**Figure 2:**
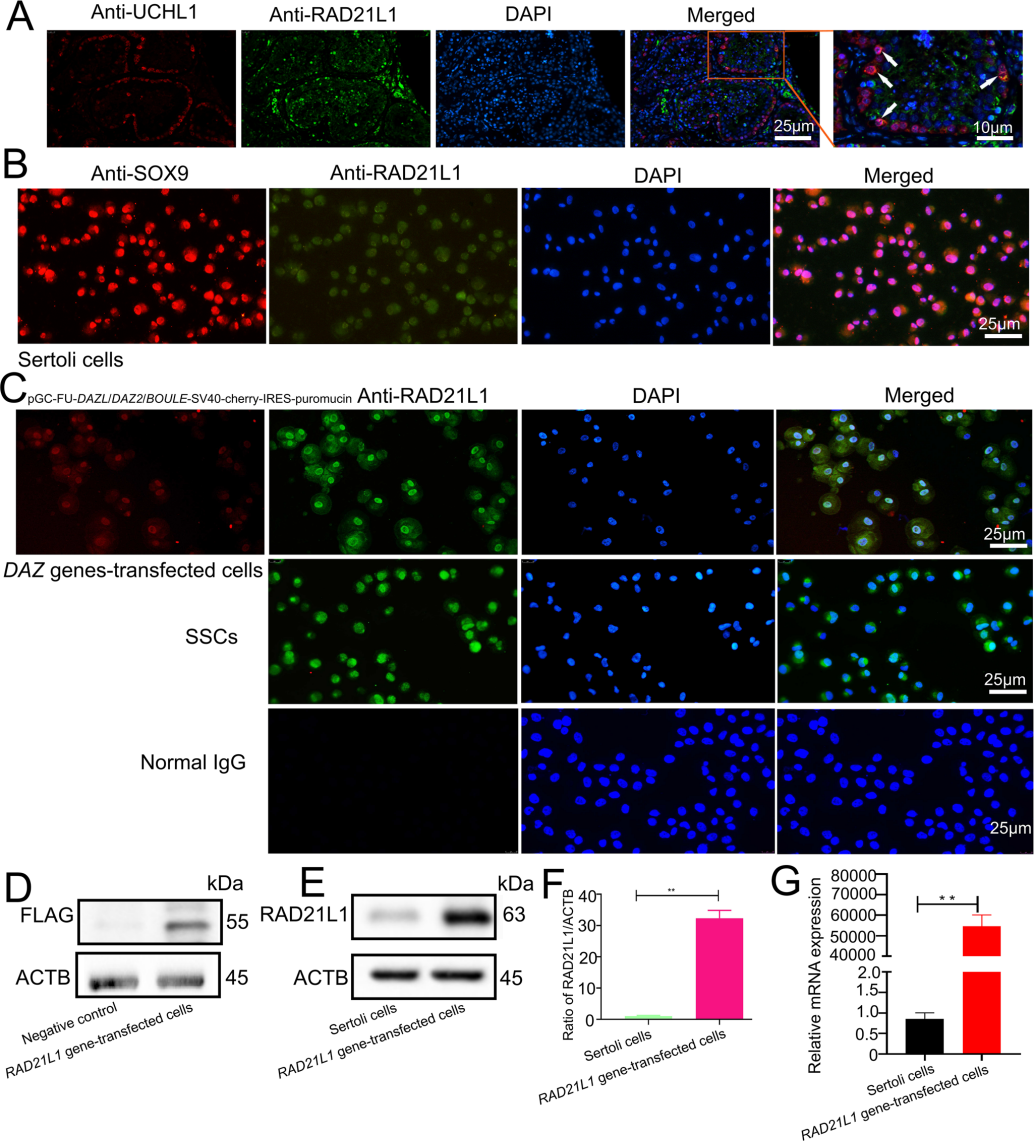
*RAD21L1* is expressed in human SSCs and it can be overexpressed in human Sertoli cells. A) Representative images of double immunostaining illustrated *RAD21L1* and UCHL1 co‐expression in human testicular tissues. Scale bar = 25 µm. B) Representative images of double immunostaining demonstrated that *RAD21L1* was not expressed in Sertoli cells as shown by the localization of SOX9. C) Immunohistochemistry revealed the presence of *RAD21L1* protein in *RAD21L1* gene‐transfected cells and human SSC line. Scale bars = 25 µm. D–F) Western blots were employed to detect the flag proteins and *RAD21L1* protein present in *RAD21L1* gene‐transfected cells. G) The qPCR showed that mRNA expression of *RAD21L1* was remarkably higher in *RAD21L1*‐gene transfected cells compared with human Sertoli cells. Data was presented as mean ± SD, *n* = 3; *p*‐values were calculated using Student's *t*‐test. ^**^ *p*<0. 01.

We determined the cellular localization of *RAD21L1* in human testes and its role in directing Sertoli cells reprogramming into human SSCs. Interestingly, our double immunostaining illustrated that *RAD21L1* was co‐localized with UCHL1 in human SSCs and γH2AX in spermatocytes in seminiferous tubules of human testes (**Figure** **2**A; Figure S1A,B, Supporting Information). Our double immunostaining demonstrated that *RAD21L1* was not expressed in Sertoli cells, as evidenced by the localization of SOX9 in these cells (Figure 2B). Moreover, immunocytochemistry revealed the localization of *RAD21L1* in the nuclei of *DAZ* genes‐transfected cells (Figure 2C, upper panel) and human SSC line (Figure 2C, middle panel).

We next probed whether *RAD21L1* could be transferred and highly expressed in human Sertoli cells using plasmids overexpressing *RAD21L1* gene fused with a flag (Figure S2, Supporting Information). The overexpressing *RAD21L1* gene plasmid was transfected to human Sertoli cells, and these cells were selected with puromycin at 3 µg mL^−1^ for 14 days. The expression of anti‐flag protein was detected by Western blots in *RAD21L1* gene‐transfected cells (Figure 2D). *RAD21L1* protein level was remarkably higher in *RAD21L1* gene ‐transfected cells than human Sertoli cells (Figure 2E,F). Our qPCR revealed that mRNA of *RAD21L1* was obviously higher in *RAD21L1* gene‐transfected cells than human Sertoli cells (Figure 2G). Collectively, these results implicate high expression levels of *RAD21L1* gene and its protein in *RAD21L1* gene‐transfected cells.

### 2.3 *RAD21L1* Gene‐Transfected Cells Are Human SSCs in Phenotype

**Figure 3:**
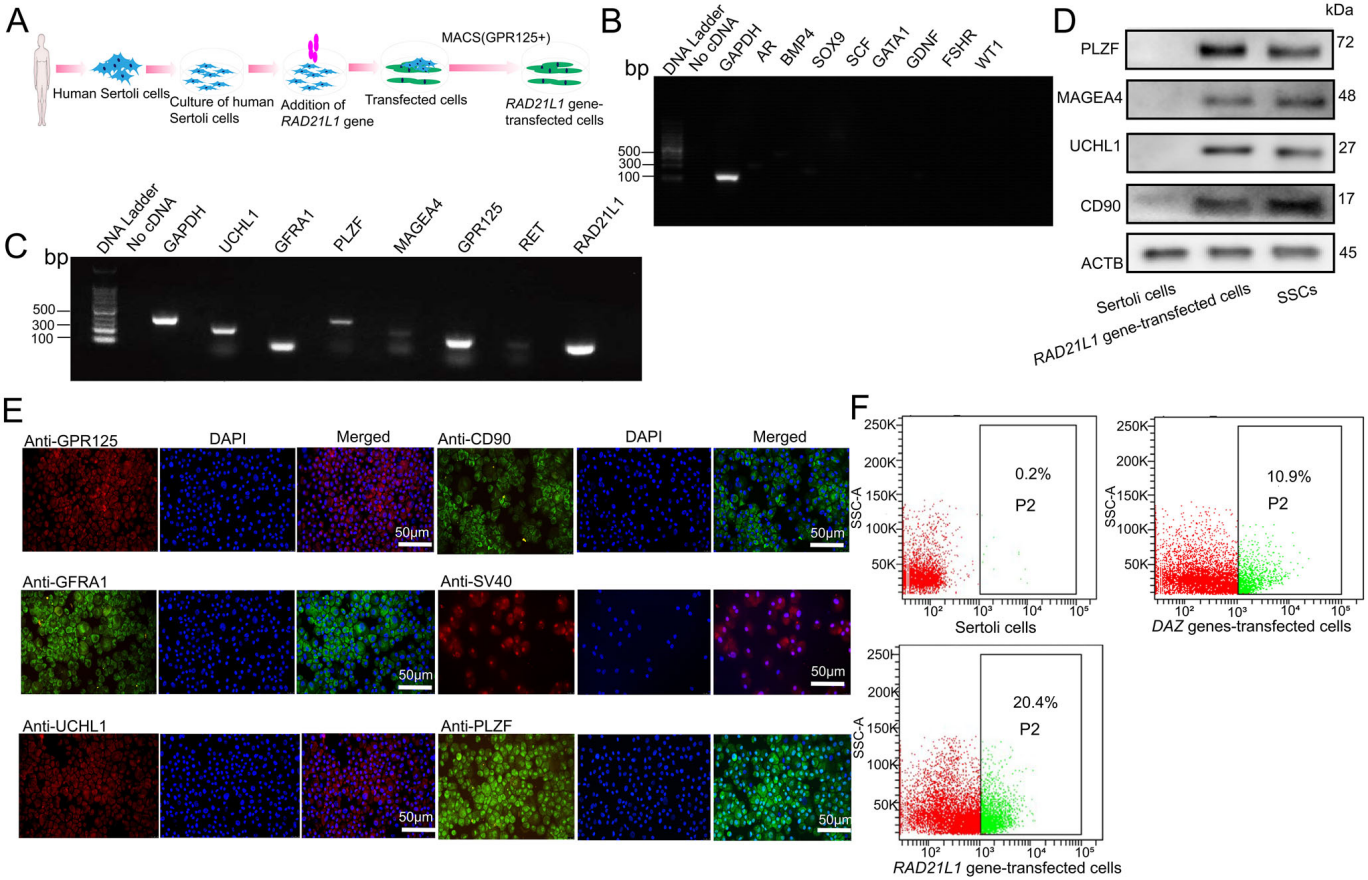
Sertoli cells overexpressing *RAD21L1* gene are human SSCs in phenotype. A) Schematic diagram illustrated reprogramming procedure of Sertoli cells to human SSCs by overexpressing *RAD21L1* gene. B) RT‐PCR showed that transcription of *SOX9*, *SCF*, *AR*, *BMP4*, *GATA1*, *GDNF*, *FSHR* and *WT1* genes was undetectable in *RAD21L1* gene‐transfected cells. C) RT‐PCR displayed transcription of *GFRA1*, *UCHL1*, *PLZF*, *MAGEA4*, *GPR125*, *RET*, and *RAD21L1* genes in *RAD21L1* gene‐transfected cells. *GAPDH* was used as a control of loading RNA. D) Western blots were employed to detect the PLZF, MAEGEA4, UCHL1, and CD90 proteins present in *RAD21L1* gene‐tansfected cells. E) Immunocytochemistry revealed GPR125, GFRA1, UCHL1, CD90, SV40 and PLZF proteins in *RAD21L1*‐gene transfected cells. F) The CD90^+^ cells were isolated from *DAZ* genes‐transfected cells and *RAD21L1* gene‐transfected cells by FACS.

We asked whether *RAD21L1* overexpression could reprogram Sertoli cells into human SSCs. Magnetic‐activated cell sorting (MACS) was employed to select GPR125^+^ cells from *RAD21L1* gene‐transfected cells (**Figure** **3**A). The expression of marker genes for Sertoli cells, including *AR*, *BMP4*, *SOX9*, *SCF*, *GATA1*, *GDNF*, *FSHR*, and *WT1*, was undetectable in *RAD21L1* gene‐transfected cells (Figure 3B). In contrast, transcripts of *UCHL1*, *GFRA1*, *PLZF*, *MAGEA4*, *GPR125*, and *RET*, hallmarks for human SSCs and spermatogonia, and *RAD21L1* gene were detected in *RAD21L1* gene‐transfected cells (Figure 3C). Western blots indicated that PLZF, UCHL1, MAGEA4, and CD90 (THY1) proteins were expressed in *RAD21L1* gene‐transfected cells (Figure 3D). Moreover, immunostaining illustrated that *RAD21L1* gene‐transfected cells were stained positively for a number of proteins for human SSCs, including GPR125, UCHL1, GFRA1, CD90, and PLZF (Figure 3E). Together, these results suggest that *RAD21L1* gene‐transfected cells are human SSCs in phenotype.

To compare the efficiency of reprogramming *DAZ* genes‐transfected cells and *RAD21L1* gene‐transfected cells from human Sertoli cells, fluorescence‐activated cell sorting (FACS) was performed using anti‐CD90 (also named THY1), a specific hallmark for human SSCs. Interestingly, we found that 10.9% of cells were positive for CD90 in *DAZ* genes‐transfected cells (Figure 3F), and notably, 20.4% of cells were stained positively for CD90 in *RAD21L1* gene‐transfected cells (Figure 3F). This data reflects a higher efficiency of reprogramming of human Sertoli cells by overexpression of only *RAD21L1* than three *DAZ* genes. We further conducted an analysis of the status of *DAZ* genes‐transfected cells in the context of *RAD21L1* silencing that led to the decrease in stemness characteristics in these cells (Figure S3A–E, Supporting Information).

### 2.4 *RAD21L1 G*ene‐Transfected Cells Assume Normality in Chromosomes and Without Y Chromosome Gene Microdeletions

**Figure 4:**
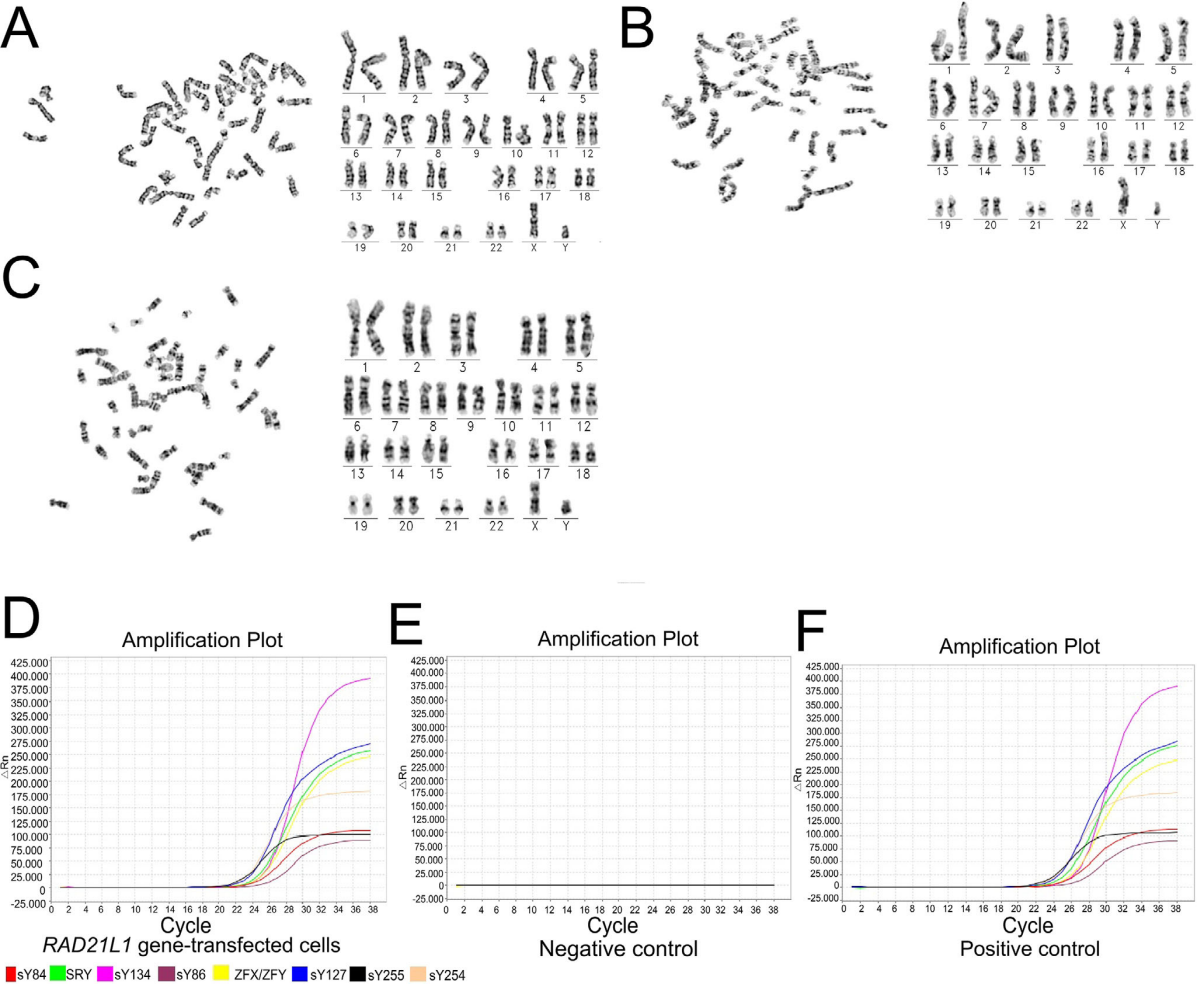
*RAD21L1* gene‐transfected cells have normal chromosomes and no Y chromosome gene microdeletions. A–C) Cytogenetic analysis revealed 23 pairs of chromosomes in *RAD21L1* gene‐transfected cells. D) Multiplex qPCR displayed the transcripts of *sY127*, *sY134*, *sY86*, *sY84*, *sY254*, *sY255*, *SRY*, and *ZFX/Y* in *RAD21L1* gene‐transfected cells. E) Water substituted for DNA and was used as a negative control. F) DNA derived from normal human blood was employed as a positive control.

Gene transferring into cells can cause unusual karyotype. We found that 23 pairs of chromosomes were present in 100% of *RAD21L1* gene‐transfected cells, and there was no chromosomal numerical aberration or unbalanced translocation in these cells (**Figure** **4**A–C), reflecting that *RAD21L1* gene‐transfected cells have normal chromosomes. As shown by multiplex PCR assay, the transcript of *sY86*, *sY84*, *sY127*, *sY134*, *sY254*, *sY255*, *SRY*, and *ZFX/Y* were detectable in *RAD21L1* gene‐transfected cells (Figure 4D). No DNA template but with water was employed as a negative control (Figure 4E), whereas normal blood DNA served as a positive control (Figure 4F). Taken together, our results indicate that *RAD21L1* gene‐transfected cells have safety since they have normal chromosomes and no Y chromosome microdeletions.

### 2.5 *RAD21L1 G*ene‐Transfected Cells Can Be Differentiated into Spermatocytes In Vitro

**Figure 5:**
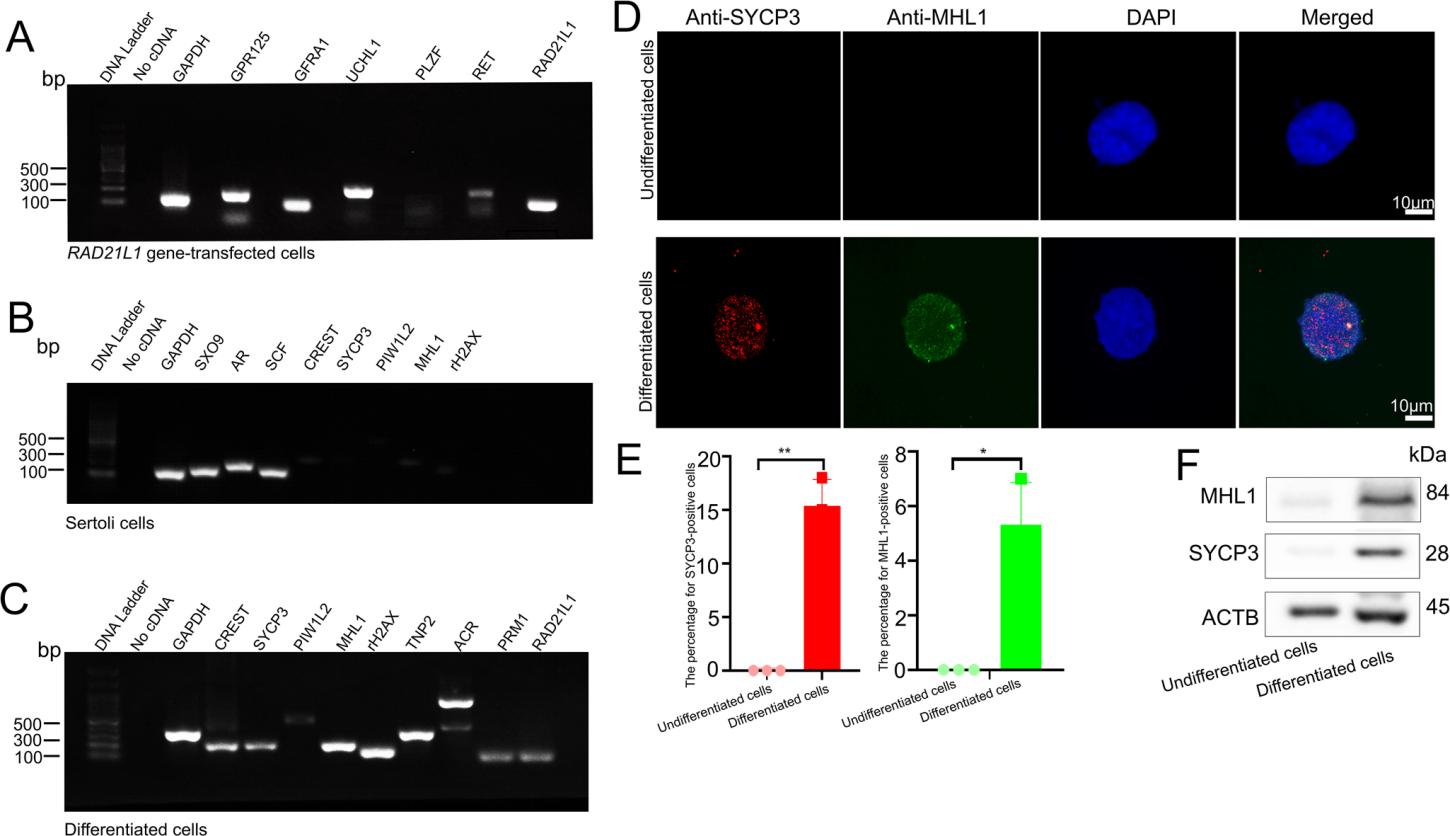
*RAD21L1* gene‐transfected cells are coaxed to differentiate into spermatocytes in vitro. A) RT‐PCR showed the transcription of *GFRA1*, *GPR125*, *UCHL1*, *PLZF*, *RET*, and *RAD21L1* in *RAD21L1* gene‐transfected cells. B) RT‐PCR displayed transcripts of *SOX9*, *AR*, *SCF*, *CREST, SYCP3, PIWIL2, MHL1*, and *rH2AX* in human primary Sertoli cells. C) RT‐PCR demonstrated transcription of *CREST*, *SYCP3*, *PIWIL2*, *MLH1*, *rH2AX*, and *RAD21L1* in the differentiated cells obtained from *RAD21L1* gene‐transfected cells for 14 days of induction. *GAPDH* was employed as a loading control of total RNA. D,E) Meiotic spread assay illustrated the co‐expression of SYCP3 (red fluorescence) MLH1 (green fluorescence) in *RAD21L1* gene‐transfected cells after 14 days of induction. DAPI with blue fluorescence was used to label nuclei of cells. Data were presented as mean ± SD, *n* = 3; *p*‐values were calculated using Student's *t*‐test.^*^ *p*<0. 05; ^**^ *p*<0. 01. F) Western blots revealed that SYCP3 and MHL1 proteins were detected in the differentiated cells from *RAD21L1* gene‐transfected cells.

We examined the differentiation potential of *RAD21L1* gene‐transfected cells. Transcripts of *GFRA1*, *UCHL1*, *PLZF*, *GPR125*, and *RET*, hallmarks for human SSCs, and *RAD21L1* were detected in *RAD21L1* gene‐transfected cells (**Figure** **5**A), reflecting the identifies of these cells as human SSCs. Transcription of *SOX9*, *AR*, and *SCF* (markers of human Sertoli cells) was detected in human primary Sertoli cells, and *CREST, SYCP3, PIWIL2, MHL1, rH2AX* (hallmarks for spermatocytes) were undetectable in these cells (Figure 5B). Intriguingly, *SYCP3*, *CREST*, *MLH1*, *PIWIL2*, and *rH2AX* genes were observed in *RAD21L1* gene‐transfected cells after 14 days of culture with the conditioned medium (Figure 5C). Immunostaining was performed to detect SYCP3 and MLH1 expression in the differentiated cells derived from *RAD21L1* gene‐transfected cells. SYCP3‐ and MCHL1‐positive cells (Figure 5D,E) could be seen in *RAD21L1* gene‐transfected cells after 14 days of induction as shown by meiotic spreads, which implicates that they were spermatocytes in phenotype. Similarly, Western blots indicated that SYCP3 and MHL1 proteins were expressed at high levels in the differentiated cells (Figure 5F). Collectively, these results implicate that *RAD21L1* gene‐transfected cells derived from human Sertoli cells have the potential of differentiation into spermatocytes.

### 2.6 *RAD21L1* Reprograms Sertoli Cells into Human SSCs via Stem Cell Maintenance and DNA Methylation

**Figure 6:**
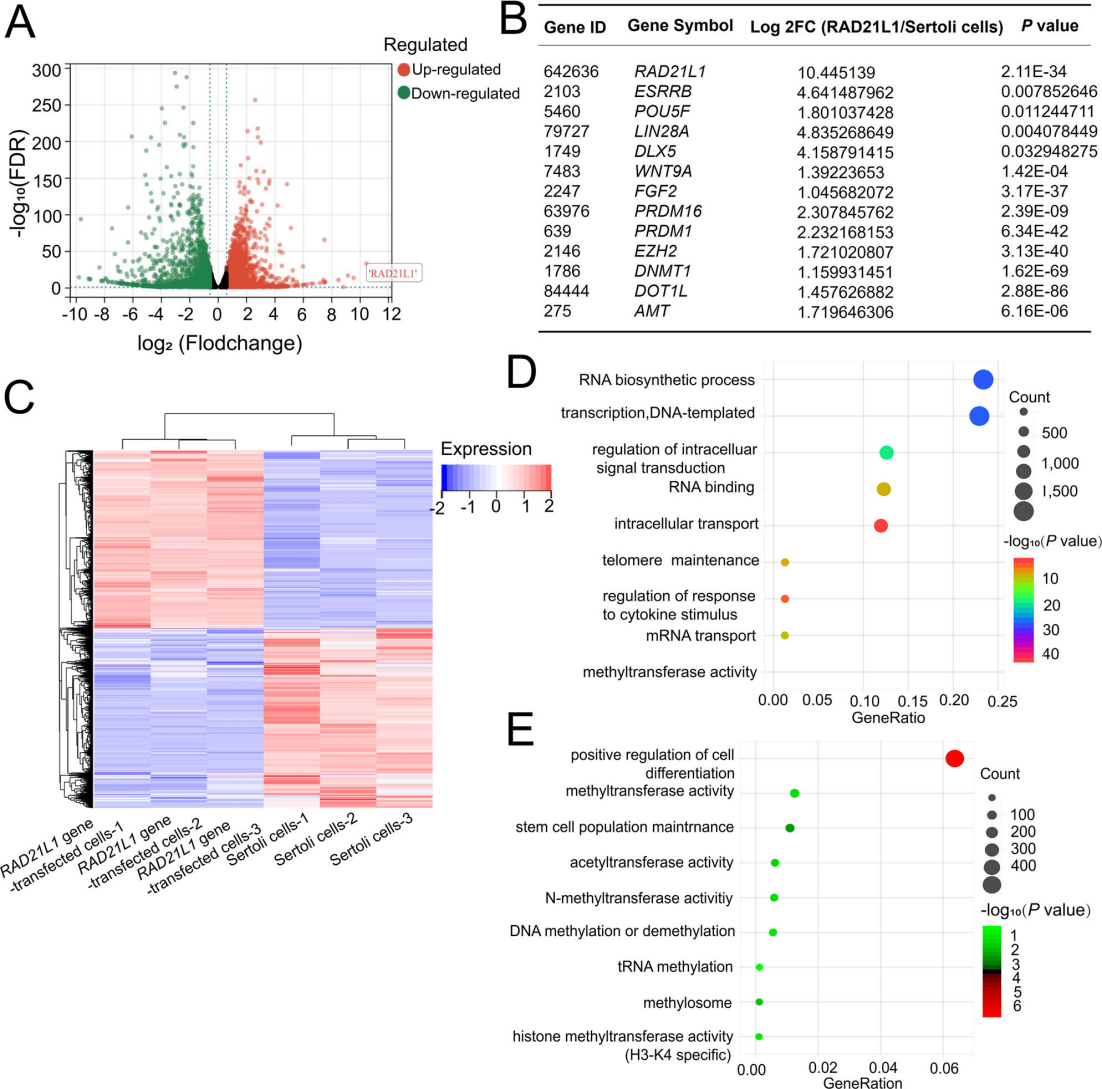
*RAD21L1* reprograms Sertoli cells into human SSCs via DNA methylation and stemness maintenance pathways. A) Volcano plot demonstrated the DEGs between *RAD21L1* gene‐transfected cells and human Sertoli cells. B) RNA‐seq indicated that *RAD21L1* gene was highly expressed in *RAD21L1* gene‐transfected cells. C) Hierarchical clustering showed the DEGs between *RAD21L1* gene‐transfected cells and Sertoli cells. Notes: Red color represented a relatively higher expression level of genes, while blue color indicated a relatively lower expression level of genes. D) KEGG pathway analysis of the DEGs between *RAD21L1* gene‐transfected cells and human Sertoli cells. Items with larger bubbles had more DEGs. Bubble colors were changed from purple, blue, green to red, which implicated the smaller the enrichment *P* value. E) GO assay displayed top 9 enrichment functional terms.

RNA‐seq was used to compare global transcriptomic profiles between *RAD21L1* gene‐transfected cells and human Sertoli cells. There were 8796 DEGs between *RAD21L1* gene‐transfected cells and human Sertoli cells. In total, 4377 genes were upregulated whereas 4419 genes were downregulated in *RAD21L1* gene‐transfected cells compared to human Sertoli cells as shown by volcano plot and heat cluster map and (**Figure** **6**A). *RAD21L1* and *DNMT1* as well as several SSC marker genes, including *POU5F1* (*OCT4*), *EZH2*, and *LIN28A* were upregulated by *RAD21L1* overexpression (Figure 6B). Heat map demonstrated different transcription profiles between *RAD21L1* gene‐transfected cells and human Sertoli cells (Figure 6C), and our heat map analysis indicated no significant difference in transcripts of *UCHL1*, *THY1*, *GFRA1* and *PCNA* between *RAD21L1* gene‐transfected cells and human SSC line (Figure S4, Supporting Information). *RAD21L1* gene‐transfected cells had similar transcriptome with *DAZ* genes‐transfected cells (Figure S5A, Supporting Information), and the transcripts of *UCHL1* and *THY*1, markers for human SSCs, were expressed at higher levels in *RAD21L1* gene‐transfected cells from human Sertoli cells than *DAZ* genes‐transfected cells (Figure S5B,C, Supporting Information). We revealed that DEGs were mainly enriched in stem cell population maintenance, DNA methylation or demethylation, and methyltransferase activity, as shown by KEGG and GO analyses (Figure 6D,E). These data demonstrate that *RAD21L1* can reprogram Sertoli cells into human SSCs via stem cell maintenance and DNA methylation pathways.

**Figure 7:**
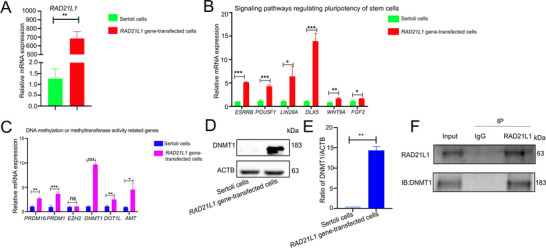
*RAD21L1* transits Sertoli cells to human SSCs through DNMT1. A) The qPCR revealed that mRNA expression of *RAD21L1* was remarkably higher in *RAD21L1* gene‐transfected cells compared to human Sertoli cells. B) The qPCR demonstrated that transcripts of signaling pathways and stem cell population maintenance genes, including *ESRRB*, *POU5F1*, *LIN28A*, *DLX5*, *WNT9*, and *FGF2* in *RAD21L1* gene‐transfected cells. C) The qPCR showed that mRNA expression of DNA and histone methylation or methytransferase activity genes, including *DNMT1*, *DOT1L, PRDM16*, *PRDM1*, *EZH2*, and *AMT* in *RAD21L1* gene‐transfected cells. D,E) Western blots illustrated DNMT1 protein level in *RAD21L1* gene‐transfected cells and human Sertoli cells. Data were presented as mean ± SD, *n* = 3; *p*‐values were calculated using Student's *t*‐test. ^**^ *p*<0. 01. F) Co‐IP demonstrated the interaction of DNMT1 with *RAD21L1* in *RAD21L1* gene‐transfected cells.

*RAD21L1* transcription between *RAD21L1* gene‐transfected cells and human Sertoli cells identified by RNA‐seq was further verified by qPCR (**Figure** **7**A). Several genes identified by RNA‐seq to be upregulated by *RAD21L1*, for example, signaling pathways regulating pluripotent stem cells, including *ESRRB*, *POU5F1* (*OCT4*), *LIN28A*, *DLX5*, *WNT9A*, and *FGF2*, were further selected for verification by qPCR (Figure 7B). Some other genes identified by RNA‐seq, including DNA and histone methylation or methytransferase activity related genes, for example, *DNMT1*, *PRDM16*, *PRDM1*, *EZH2*, *DOT1L*, and *AMT*, were selected for validation by qPCR (Figure 7C). Notably, *DNMT1* transcript was remarkably higher in *RAD21L1* gene‐transfected cells than human Sertoli cells (Figure 7C). Western blots illustrated that DNMT1 protein was expressed at a higher level in *RAD21L1* gene‐transfected cells than human Sertoli cells (Figure 7D,E). Interestingly, our Co‐IP (Co‐immunosuppression) demonstrated that *RAD21L1* interacted with DNMT1 in *RAD21L1* gene‐transfected cells (Figure 7F). Collectively, these results reflect that *RAD21L1* transits Sertoli cells into human SSCs through DNMT1.

### 2.7 *RAD21L1* Reprograms Sertoli Cells into Human SSCs Through DNMT1

DNA methylation is involved in cell development and causes the repression of genes and transportable elements.^[^ ^16^ ^]^ DNA methyltransferases (DNMTs) have been reported to reprogram cell fate determinations by causing genome to undergo demethylation and remethylation.^[^ ^17^ ^]^ DNA methylation is related to cell reprogramming during human primordial germ cell development.^[^ ^18^ ^]^ We found that levels of DNA and histone methylation or methytransferase activity related genes, including *DNMT1*, *PRDM16*, *PRDM1*, *EZH2*, *DOT1L*, and *AMT*, were upregulated in *RAD21L1* gene‐transfected cells compared to human Sertoli cells by RNA‐seq and qPCR (Figure 7C). Notably, methyltransferase DOT1L is required for maintaining mouse SSC self‐renewal,^[^ ^19^ ^]^ and we proposed that DOT1L was involved in reprogramming Sertoli cells to human SSCs based upon our data.

**Figure 8:**
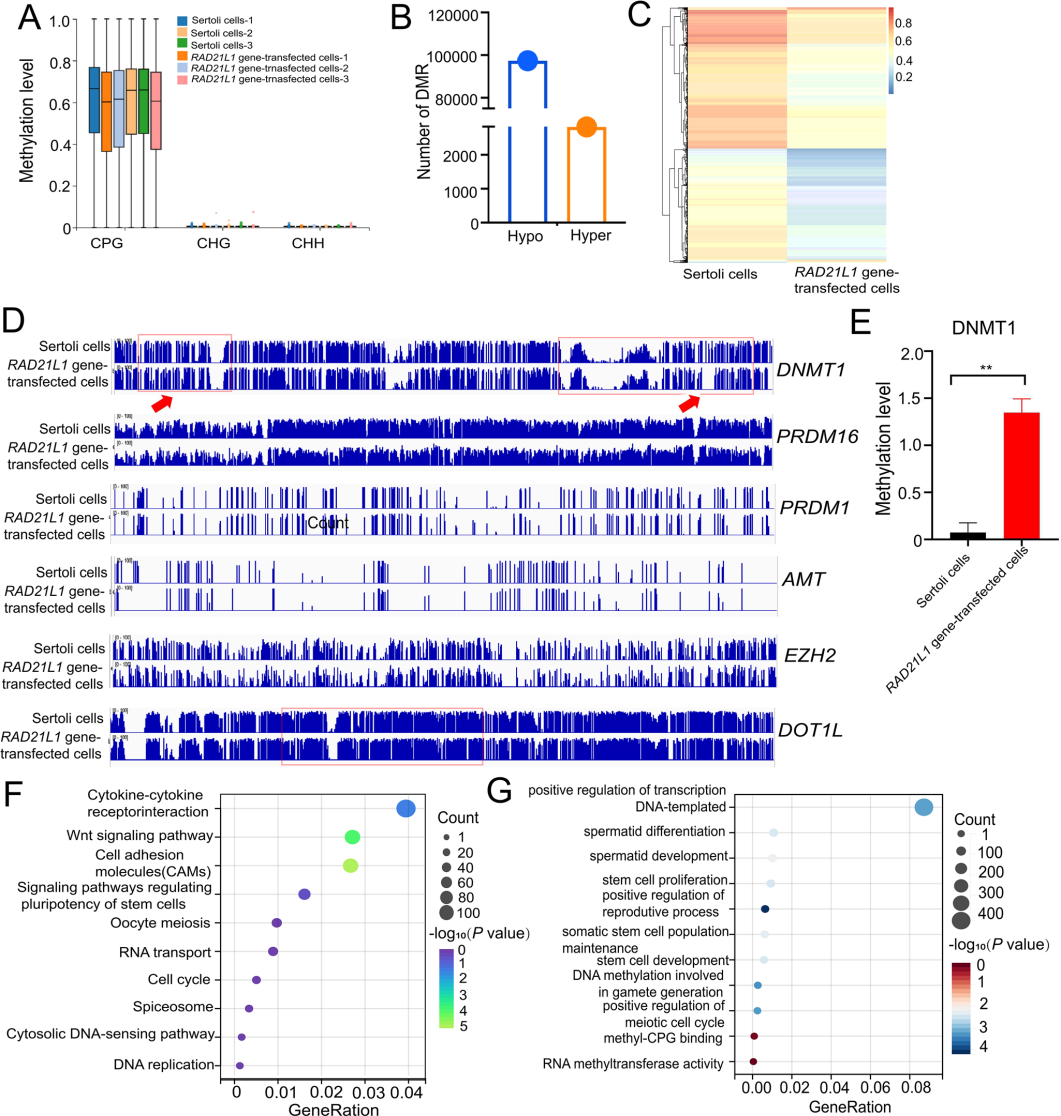
*RAD21L1* directs human Sertoli cells to human SSCs through DNA methylation. A) Map illustrated that DNA methylation existed at CPG sites, including genome‐wide methylation status. B) Numbers of differentially methylated regions (DMRs) between *RAD21L1* gene‐transfected cells and human Sertoli cells. C) Hierarchical clustering showed the DMRs in *RAD21L1*‐gene transfected cells and human Sertoli cells. Notes: Red color represented a relatively higher expression level of genes, whereas blue color indicated a relatively lower expression level of genes. D,E) IGV analysis demonstrated that chromosomal loci, including *DNMT1*, *PRDM16*, *PRDM1*, *AMT*, *EZH2*, and *DOT1L*, with *DNMT1* having the greatest variation in *RAD21L1* gene‐transfected cells compared with human Sertoli cells. Result was shown as mean ± SD, *n* = 3; *p*‐values were calculated using Student's *t*‐test. ^**^ *p*<0. 01. F) KEGG pathway analysis of the DEGs in *RAD21L1* gene‐transfected cells and human Sertoli cells. The items with larger bubbles contained more DEGs. The bubble colors were changed from purple, blue, green to red, which reflected the smaller the enrichment *P* value. G) GO analysis showed top 11 enrichment functional terms.

Next, we conducted WGBS to compare the differentially methylated regions (DMRs) between *RAD21L1* gene‐transfected cells and human Sertoli cells. Significantly, we revealed that DNA methylation occurred at CPG sites because of genome‐wide methylation status (**Figure** **8**A). There were 100226 DMRs between *RAD21L1* gene‐transfected cells and human Sertoli cells (Figure 8B,C). Our IGV analysis illustrated the chromosomal loci, including *DNMT1*, *PRDM16*, *PRDM1*, and *EZH2*, and *DNMT1* had the most significant methylation site variations in *RAD21L1* gene‐transfected cells compared to human Sertoli cells (Figure 8D,E), which was in agreement with our qPCR data (Figure 7C). The KEGG and GO analyses demonstrated that DMRs were involved in regulating stem cell pluripotency, stem cell proliferation, stem cell development, DNA methylation, and methyl‐CpG binding (Figure 8F,G). Considered together, our results implicate that *RAD21L1* regulates Sertoli cells’ reprogramming into human SSCs through DNA methylation, especially via DNMT1.

### 2.8 *RAD21L1* Mutation Decreases Stemness Maintenance of Human SSCs from Sertoli Cells and Expression Levels of DNMT1 Transcription and Protein

**Figure 9:**
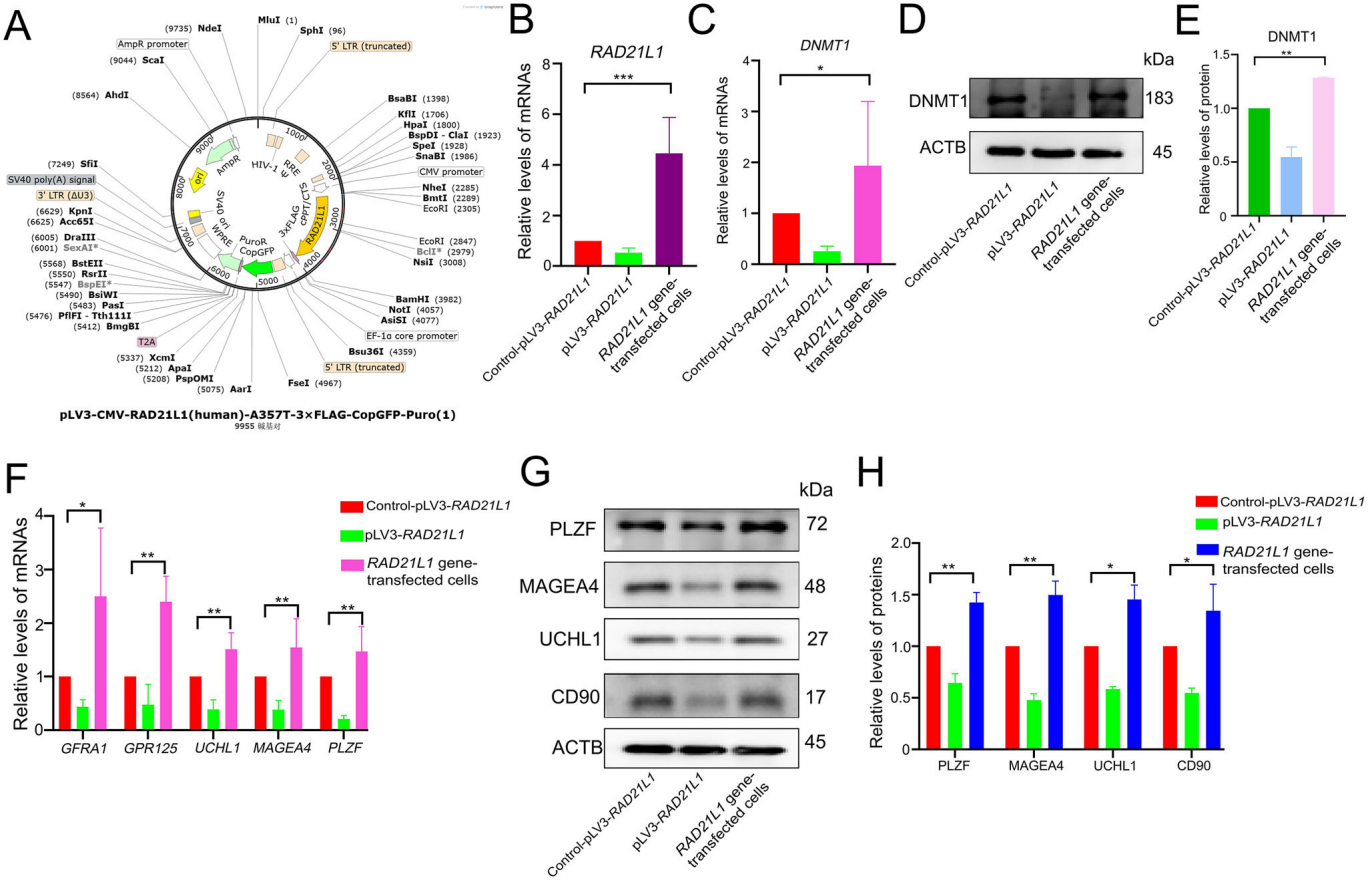
*RAD21L1* mutation inhibits the reprogramming human Sertoli cells into SSCs by DNMT1. A) The diagram showed the lentivirus *RAD21L1* mutation plasmid map. B) Real‐time PCR displayed *RAD21L1* mRNA in the *RAD21L1* gene‐transfected cells with the transfection of the lentivirus *RAD21L1* mutation plasmid. C) The qPCR showed *DNMT1* mRNA in the *RAD21L1* genes‐transfected cells without or with the treatment of *RAD21L1* mutation. D,E) Western blots exhibited DNMT1 protein alteration in the *RAD21L1* genes‐transfected cells by *RAD21L1* mutation. F) The qPCR showed the changes of *GFRA1*, *GPR125*, *UCHL1*, *MAGEA4* and *PLZF* transcripts in the *RAD21L1* genes‐transfected cells by *RAD21L1* mutation. G,H) Western blots demonstrated the protein levels of PZLF, MAGEA4, UCHL1, and CD90 in the *RAD21L1* genes‐transfected cells without or with the treatment of *RAD21L1* mutation. Results in (B), (C), (E), (F), and (H) were presented as mean ± SD, *n* = 3; *p*‐values were calculated using Student's *t*‐test.^*^ *p*<0. 05; ^**^ *p*<0. 01;^**^ *p*<0. 001.

We constructed and employed the lentivirus *RAD21L1* mutation plasmid (**Figure** **9**A). Our real‐time PCR revealed that *RAD21L1* mRNA was reduced in *RAD21L1* gene‐transfected cells after transfecting the lentivirus *RAD21L1* mutation plasmid (Figure 9B). Intriguingly, we observed the decreases in DNMT1 expression at both mRNA and protein by the lentivirus *RAD21L1* mutation plasmid (Figure 9C–E). We asked whether *RAD21L1* mutation influences the reprogramming of Sertoli cells into human SSCs. Our real‐time PCR revealed remarkable reduction in transcripts of *GFRA1*, *GPR125*, *UCHL1*, *MAGEA4* and *PLZF* in *RAD21L1* gene‐transfected cells after the transfection of the lentivirus *RAD21L1* mutation plasmid (Figure 9F). Western blots demonstrated the decreases in the protein levels of GFRA1, GPR125, UCHL1, MAGEA4 and PLZF in these cells by *RAD21L1* mutation (Figure 9G,H; Figure S3D,E, Supporting Information). Collectively, these results implicate that *RAD21L1* mutation leads to the decreases in the stemness maintenance of human SSCs from Sertoli cells and expression levels of DNMT1 transcription and translation.

### 2.9 *RAD21L1* Mutations Are Associated With Male Infertility

**Figure 10:**
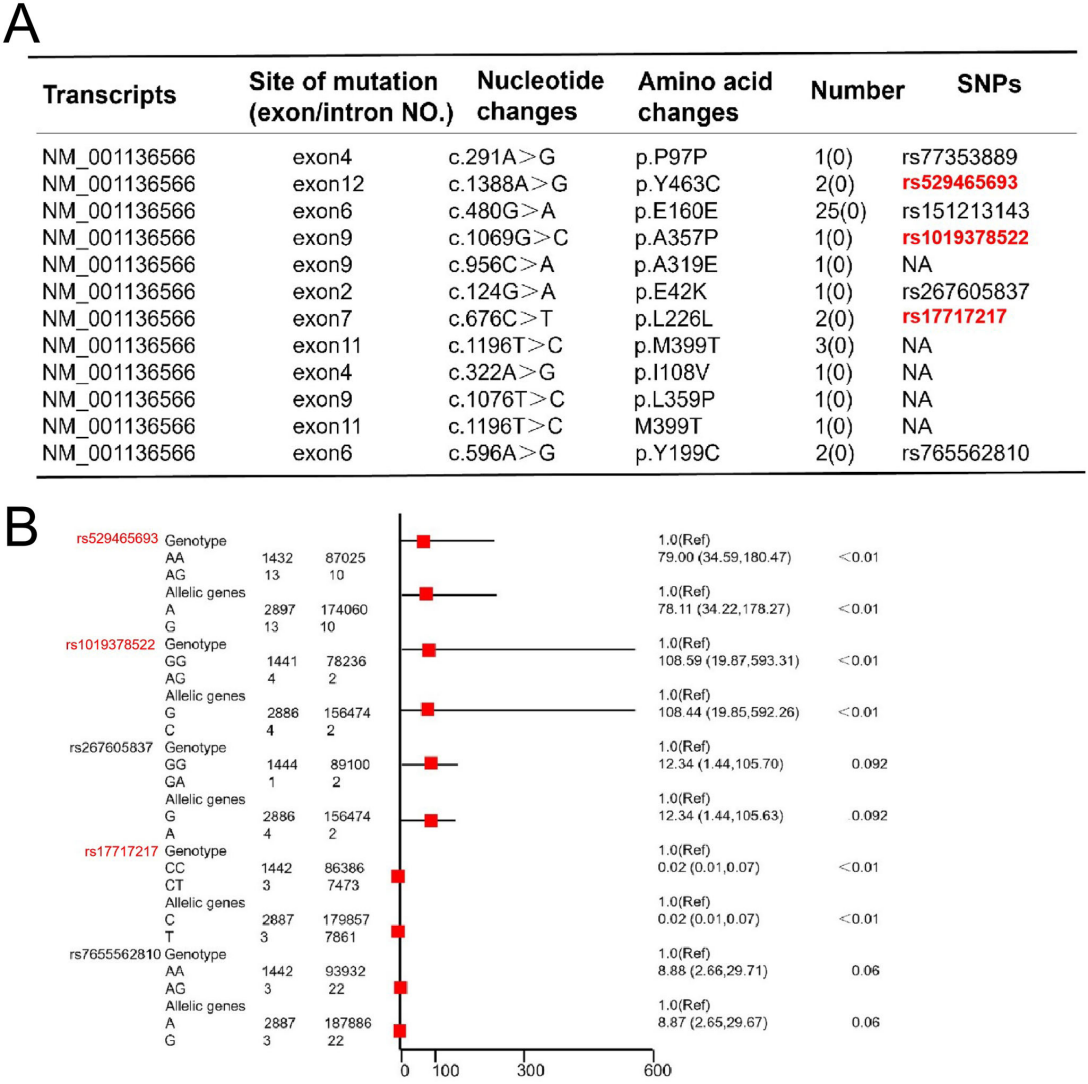
*RAD21L1* mutations are associated with NOA. A) The *RAD21L1* gene mutation distribution by WES in 1455 NOA patients with spermatogenesis failure. B) The forest map illustrated that *RAD21L1* mutations were related to the risk of spermatogenesis disorders. The chi‐square test and logistic regression analysis were employed for *RAD21L1* mutations.

Finally, we asked whether *RAD21L1* was positively associated with spermatogenic failure. Wholeexome sequencing (WES) identified 41 *RAD21L1* mutations in 1455 NOA patients (2.817% mutation rate, Table S1, supporting information). The *RAD21L1* mutations were shown below: 25 *RAD21L1* mutations were at exon6: c.480G>A (p.E160E), while 3 *RAD21L1* mutations were at exon11: c.1196T>C (p.M399T). There were 2 *RAD21L1* mutations at exon12: c.1388A>G(p.Y463C), 2 *RAD21L1* mutations at exon7: c.676C>T(p.L226L), 2 *RAD21L1* mutations at exon6: c.596A>G(p.Y199C), 1 *RAD21L1* mutation at exon4: c.291A>G(p.P97P), 1 *RAD21L1* mutation at exon9: c.1069G>C(p.A357P), 1 *RAD21L1* mutation at exon9: c.956C>A(p.A319E), 1 *RAD21L1* mutation at exon2: c.124G>A(p.E42K), 1 *RAD21L1* mutation at exon4: c.322A>G(p.I108V), 1 *RAD21L1* mutation at exon9: c.1076T>C(p.L359P), and 1 *RAD21L1* mutation at exon11: c.1196T>C (M399T) (**Figure** **10**A).

We further assessed harmfulness of *RAD21L1* mutation sites in NOA patients. Interestingly, numerous *RAD21L1* variations were seen to be damaged, especially 1069G>C(p.A357P). As examined by a chi‐square test and logistic regression analysis of *RAD21L1* mutation sites, rs529465693 (*p*<0.001), rs1019378522 (*p*<0.001), and rs17717217 (*p*<0.001) of *RAD21L1* were statistically significant (Figure 10B). Notably, genotype and allele frequencies of rs529465693, rs1019378522, and rs17717217 loci of *RAD21L1* in spermatogenesis failure patients were higher when compared to normal population from gnomAD. Collectively, our results reflect that these *RAD21L1* variation loci were correlated to the risk of spermatogenesis failure.

## 3 Discussion

Human iPS cells have been utilized to generate germ cells,^[^ ^20^ ^]^ and we have reported that *DAZ* family three genes can transmit Sertoli cells into human SSCs that exhibit phenotypic characteristics of primary human SSCs.^[^ ^6^ ^]^ Notably, we have for the first time demonstrated that *RAD21L1* is involved in Sertoli cell reprogramming into human SSCs by overexpression of *DAZ* family genes. To our knowledge, this is the first study highlighting overexpression of *RAD21L1* alone is more efficient to transmit Sertoli cells into human SSCs with similar phenotypes, which is more convenient and much safer compared to three genes of *DAZ* family. Interestingly, human SSCs derived from *RAD21L1* overexpression could be differentiated into spermatocytes with the conditioned medium in vitro. This finding represents an exciting means of obtaining male germ cells in vitro and has important implications for treating azoospermia.

Furthermore, we identified phenotypic characteristics of human SSCs reprogrammed in vitro from Sertoli cells via overexpression of *RAD21L1* gene. First of all, RT‐PCR and immunocytochemistry demonstrated that numerous human SSC marker genes, including *GFRA1*, *PLZF*, *GPR125*,*UCHL1*, and *RET*, were expressed in *RAD21L1* gene‐transfected cells. Retinoic acid (RA) can induces mouse SSCs to differentiate into spermatocytes,^[^ ^20^ ^]^ and we have shown that RA and stem cell factor (SCF) can coax human SSCs to give rise to spermatocytes and spermatids.^[^ ^21^ ^]^ In this study, SYCP3 and MLH1 were found to be expressed in *RAD21L1* gene‐transfected cells after two weeks of culture in the conditioned medium, which implies the generation of spermatocytes. To assess the safety caused by gene transfection, karyotype and Y chromosome microdeletions were examined in *RAD21L1* gene‐transfected cells, and we observed that these cells exhibited normal chromosomal characteristics. Our multiplex PCR revealed no Y chromosome microdeletions in *RAD21L1* gene‐transfected cells. As such, our transferring exogenous *RAD21L1* gene into Sertoli cells causes no chromosomal change for human SSCs, which was safer compared to previous reports showing changes in chromosomes following gene overexpression.^[^ ^22^ ^]^ Secondly, our RNA‐seq highlights that transcriptome of *RAD21L1* gene‐transfected cells was distinct from human Sertoli cells. In addition, we revealed that DEGs were enriched for DNA methylation, demethylation, and methyltransferase activity, and our qPCR demonstrated that DNA and histone methylation or methytransferase activity related genes were highly expressed in *RAD21L1* gene‐transfected cells. Thirdly, we elucidated the molecular mechanism underlying the reprogramming of Sertoli cells into human SSCs by *RAD21L1*. Our RNA‐seq revealed high levels of DNA and methylation or histone methyltransferase activity‐related genes, especially *DNMT1* and *DOT1L*. DNA methylation plays a crucial role in silencing retrotransposon and it is essential for mammalian cell development.^[^ ^23^ ^]^ It has been reported that DNA methylation undergoes highly dynamic changes during embryogenesis.^[^ ^24^ ^]^ Reprogramming abnormalities are associated with human embryonic arrest.^[^ ^25^ ^]^ Intriguingly, we found that *RAD21L1* mutation impaired DNMT1 expression and the stemness maintenance of human SSCs from Sertoli cells. It has been reported that DOT1L is required for maintaining SSC self‐renewal.^[^ ^19^ ^]^ Our WGBS analysis illustrated that *RAD21L1* regulates fate decisions of Sertoli cell reprogramming into human SSCs through DNA methylation. These findings highlight that uncovering the reprogramming mechanisms of somatic cells to male germ cells is of unusual significance for reproductive medicine and regenerative medicine. Moreover, we identified 41 mutations of *RAD21L1* gene in 1455 NOA patients using WES and variations of *RAD21L1* loci were positively associated with spermatogenesis disorders.

**Figure 11:**
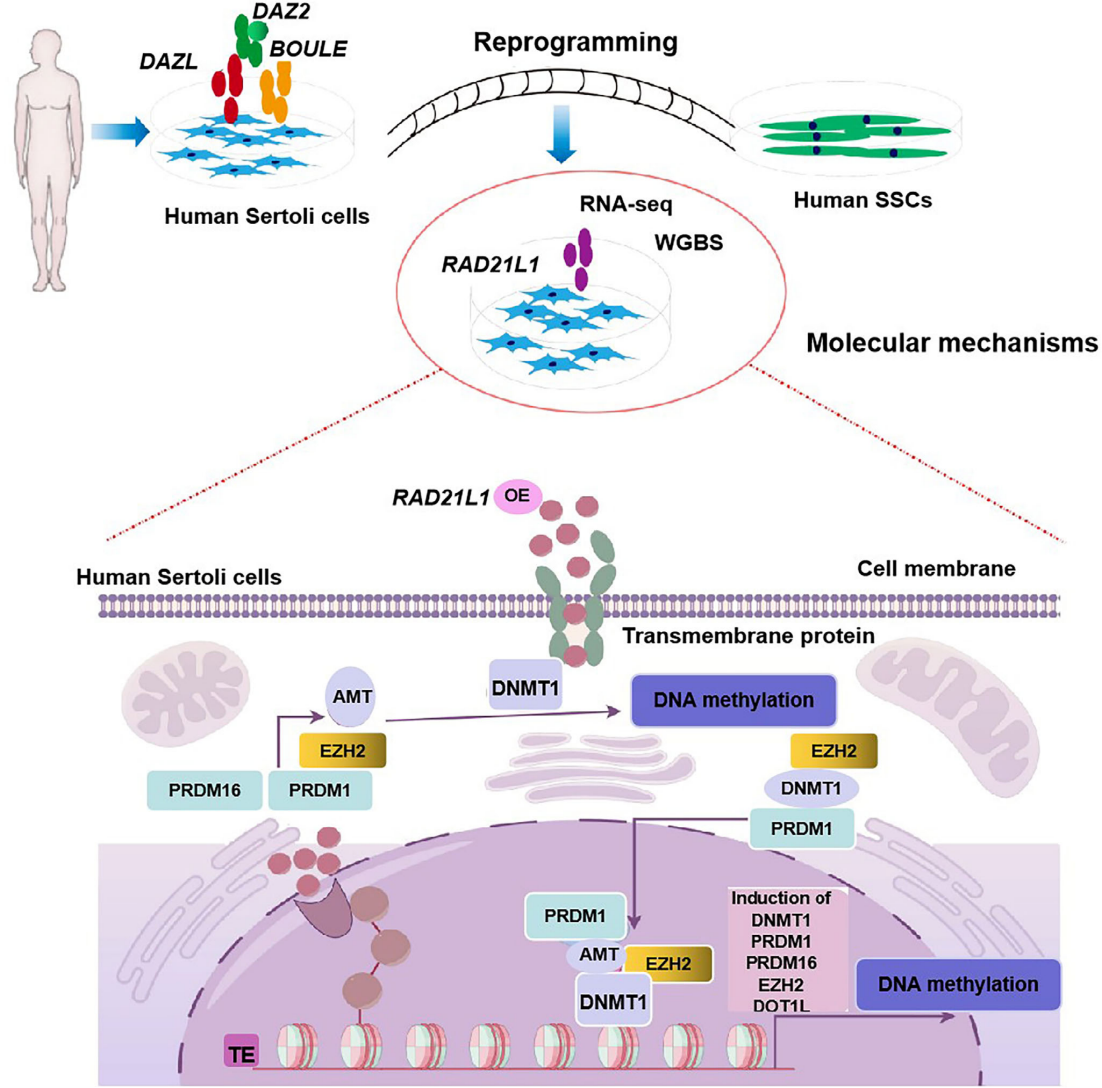
Schematic diagram illustrates the function and mechanism of *RAD21L1* in transmitting human Sertoli cells to SSCs, as well as its mutations with the risk of NOA. *RAD21L1* is upregulated by overexpressing *DAZ* family genes, and *RAD21L1* is essential for reprogramming Sertoli cells into human spermatogonial stem cells through DNA methylation. *RAD21L1* mutations are correlated with the risk of spermatogenesis failure.

In summary, we have unveiled the reprogramming mechanism of Sertoli cells into human male germline stem cells by overexpressing *DAZ* family three genes via *RAD21L1*. More significantly, we have demonstrated that overexpressing only one gene *RAD21L1* is sufficient and effective to transmit Sertoli cells to human SSCs. Furthermore, we discovered that this reprogramming can be achieved by *RAD21L1*‐mediated DNA methylation, especially DNMT1. Abnormalities in DNA methylation and *DNMT1* expression have been observed in certain NOA patients.^[^ ^26^ ^]^ Importantly, we identified that the variations of *RAD21L1* were positively associated with the risk of spermatogenesis failure. As such, *RAD21L1* is required for reprogramming Sertoli cells to human SSCs through DNA methylation and *RAD21L1* mutations are closely related to spermatogenesis failure, as we illustrated in **Figure** **11**. Therefore, our study offers a novel molecular mechanism which transmits human somatic cells (e.g., Sertoli cells, fibroblasts, and skin cells) to male germline stem cells and it provides a new approach to generate invaluable spermatozoa for NOA patients without spermatids.

## 4 Experimental Section

### Isolation and Culture of Human Sertoli Cells

Human Sertoli cells were isolated by two‐step enzymatic digestion and differential plating approach^[^ ^27^ ^]^ from testis tissues of nine OA patients with microdissection testicular sperm extraction (MTSE) or castration therapy for prostate cancer at the Third Xiangya Hospital of South‐Central China or Hunan Cancer Hospital. Human Sertoli cells were cultured with DMEM/F‐12 (Gibco, USA) supplemented with 10% fetal bovine serum (FBS) (Gibco) and 1% penicillin & streptomycin (Gibco, USA) at 34 °C under a 5% CO_2_ incubator. This study was approved by the Institutional Ethical Review Committee of Hunan Normal University (Approval number: 2021227), and an informed consent of testis tissues for research only was obtained from OA patients.

### Human SSC Line Culture

Human SSC line was set up by the team as described previously.^[^ ^28^ ^]^ This cell line was cultured with DMEM/F‐12 or StemPro‐34 SFM medium with the addition of 10% FBS and 1% penicillin streptomycin (Gibco) at 34 °C under a 5% CO_2_ incubator.

### Reprogramming Sertoli Cells into Human SSCs by Overexpressing DAZ Family Genes or *RAD21L1* Gene

The lentiviral vectors that overexpress *DAZL*, *DAZ2* and *BOULE* genes, or *RAD21L1* gene were obtained from GeneChem Co. Ltd. (Shanghai, China). The sequences of *DAZ* family genes and *RAD21L1* gene were shown in Table S2 and Figure S2 (Supporting Information). For reprogramming, 20 µL of lentiviral vectors overexpressing *DAZ* genes or *RAD21L1* gene were transfected to 1.0 × 10^5^ human Sertoli cells with 500 µL HitransG P in DMEM/F‐12 for 24 h, and they were recovered for additional 24 h. Culture medium was changed every 24 h with fresh medium with addition of 3 µg mL^−1^ puromycin for 14 days to select the positive cells.

### Fluorescence‐Activated Cell Sorting (FACS)

*DAZ* family genes‐ and *RAD21L1* gene‐transfected cells were washed with PBS thrice, and 1 × 10^6^ cells were resuspened in flow cytometry staining buffer in terms of the manufacturer's instructions. These cells were incubated with 10 µL of anti‐CD90 (THY1) or 5 µL of isotype IgG for 30 min at room temperature. The reprogramming efficiency of *DAZ* family genes‐or *RAD21L1* gene‐transfected cells was determined by FACS Canto II flow cytometry (BD Biosciences).

### RNA‐sequencing (RNA‐seq)

RNA‐seq of human *DAZ* family genes‐transfected cells, *RAD21L1* gene‐transfected cells, and human Sertoli cells was conducted by BGI‐Shenzhen (Shenzhen, China). Total RNA from cells were extracted utilizing the Trizol (Vazyme, R401‐01), and Nanodrop was employed to measure RNA purity and quality for RNA‐seq. Libraries were established by TruSeq Stranded mRNA LT Sample Prep Kit (Illumina, San Diego, CA, USA) and sequenced by Illumina HiSeq X Ten platform with generation of 150 bp paired‐end reads. Analysis of the differentially expressed genes (DEGs) was conducted utilizing the DESeq R package with thresholds as *p*<0.05 and |fold change| ≥2 for the DEGs. Further, hierarchical cluster analysis, KEGG pathway, and GO analyses of the DEGs were performed.

### Immunocytochemistry and Immunohistochemistry

*RAD21L1* gene‐transfected cells and human SSC line were placed on slides by cytospin, and 4% paraformaldehyde (PFA) was utilized to fix them for 10 min. Permeabilization of these cells was conducted using 0.25% Triton X‐100 in PBS for 10 min, and blocking of them was conducted with 5% bovine serum albumin (BSA) for 1 h. The cells were then incubated with primary antibodies (Table S3, Supporting Information) and fluorescence‐labeled IgG secondary antibodies (Table S3, Supporting Information). DAPI (4′,6‐diamidino‐ 2‐phenylindole) was used to label cellular nuclei, and immunofluorescence was observed under a fluorescence microscope (Leica, DM3000, Germany).

Sections of testicular tissues were dewaxed by xylene, and they were dehydrated with alcohol at decreasing gradients. The blocking of sections was completed with 5% BSA. Testis tissue sections were incubated first with primary antibodies (Table S3, Supporting Information) and then with secondary antibodies, including HRP‐conjugated IgG (Table S3, Supporting Information) or fluorescence‐labeled IgG (Table S3, Supporting Information). DAB staining or immunofluorescence was used to display cell immunostaining under a microscope.

### RT‐Quantitative PCR (RT‐qPCR) and RT‐PCR

Total RNA was isolated from the cells using Trizol, and cDNAs were generated by EvoM MLVRT Premix (Accurate Biology, AG11706) from 500 ng of total RNA. The qPCR reactions were performed by the SYBR Green Premix Pro TaqHs qPCR kit (Accurate Biology, AG11701) with Bio‐Rad CFX96 Connect real‐time PCR detection system. Sequences of gene primers and other information were shown in Table S4 (Supporting Information). The 2^‐ΔΔCT^ method was employed and *GAPDH* was used as housekeeping gene to compare relative expression levels of genes.

RT‐PCR was conducted using 2×Taq Master Mix (Dye Plus) (Vazyme, P222‐AA), while PCR products were separated by electrophoresis on a 2% agarose gel. The images of PCR gels were acquired by the Gel Documentation and Image Analysis System (ChampGel 5000).

### Co‐IP (Co‐Immunoprecipitation) Assay

Cells were lysed with RIPA buffer to obtain total proteins, and cell debris was removed by centrifugation at 12 000 rpm. In total, 10% of supernatant was used as input of proteins. *RAD21L1* antibody (Table S3, Supporting Information) and magnetic beads were added to cell lysate to pull down proteins. Five hundred microliter of IP buffer was used to harvest proteins, and further proteins were isolated by SDS‐PAGE gel. Western blots were conducted using antibodies as described below.

### Western Blots

RIPA lysis buffer (Beyotime, China) was used for lysing cells on ice for 30 min, and total proteins were extracted by centrifugation at 12 000 rpm. In total, 50 µg of total protein for each sample were used for the SDS‐PAGE gels, and the proteins were transferred to polyvinylidene difluoride membranes (Millipore, Germany). QuickBlock blocking buffer (Beyotime, China) was used for blockingof proteins that were incubated with primary antibodies (Table S3, Supporting Information) and then horseradish peroxidase‐conjugated secondary antibody (Table S3, Supporting Information), respectively. Mini chemiluminescence imaging system was employed to capture image of protein bands.

### Karyotype Analysis of Chromosomes

Karyotype analysis of chromosomes was performed in *RAD21L1* gene‐transfected cells with exponential growth at passage 10 pursuant to the methods as described previously.^[^ ^6^ ^]^ These cells were treated with 5 mg mL^−1^ colcemid at for 3 h, 0.075 m KCl for 25 min and fixative solution (methanol: glacial acetic acid = 3:1) for 20 min. Cell cytospin was performed, and cells were stained with Giemsa solution. Cell chromosomes were evaluated by a microscope pursuant to the recommendation of International Human Cytogenetic Nomenclature System.

### Y Chromosome Microdeletion Analysis

Microdeletions of Y chromosome in *RAD21L1* gene‐transfected cells were detected by multiplex real‐time PCR. In total, 6 specific sequence sites, namely, *sY84*, *sY134*, *sY254*, *sY86*, *sY127*, and *sY255* of azoospermia factor (AZFa, AZFb, and AZFc) on Y chromosome were determined by multiplex real‐time PCR. Genes *ZFY* and *SRY* were used as internal controls, while DNA from normal man blood served as a positive control. Water without DNA was employed as a negative control. Sequences of gene primers were shown in Table S5 (Supporting Information) and obtained from Shanghai Sangon Co., LTD.

### Differentiation of *RAD21L1* Gene‐Transfected Cells into Spermatocytes

Differentiation of *RAD21L1* gene‐transfected cells into spermatocytes was performed by culturing these cells with the conditioned medium containing DMEM/F12 supplemented with 10% KSR, 100 ng mL^−1^ BMP4, 100 ng mL^−1^ Activin A, 2×10^−6 ^mol L^−1^ retinoic acid (RA), 100 ng mL^−1^ stem cell factor (SCF), 50 ng mL^−1^ epithelial growth factor (EGF), and 10 µg mL^−1^ BPE for 14 days. Genes and proteins for spermatocytes were determined in these cells after induction of differentiation.

### Transfection of *RAD21L1* siRNAs and *RAD21L1* mutation plasmid

Transfection of *RAD21L1* gene‐transfected cells with *RAD21L1* siRNA1‐3 (Table S6, Supporting Information) and *RAD21L1* mutation plasmid (GeneChem Co. Ltd., Shanghai, China) was conducted using Lipofectamine 3000 according to the method previously described.^[^ ^29^ ^]^

### Whole‐Genome Bisulfite Sequencing

Whole‐Genome Bisulfite Sequencing was conducted by BGI‐Shenzhen (Shenzhen, China). EasyPure Genomic DNA Kit (TransGe) was employed to extract genomic DNA of cells, and bisulfite conversion was performed with the genomic DNA using the EZ DNA Methylation‐Gold Kit (Zymo Research). Sequencing libraries were established by bisulfite‐converted DNAs. For each library, 131.2 Gbp of raw data was acquired by the Illumina HiSeq X Ten sequencing system.

### Whole Exon Sequencing

Genomic DNA was extracted from the peripheral blood of 1455 NOA patients utilizing the QIAamp DNA blood midi kit (Qiagen, Hilden, Germany), and Whole Exon Sequencing (WES) of probs was conducted by Beijing Genome Institute at Shenzhen using HiSeq2000 sequencing platform (Illumina, San Diego, California, USA). WES data were aligned to NCBI GRCh37 (Reference genome Hg19) using BWA Genome Analysis Toolkit (GATK). PCR duplicates were removed and sorted by Picard (http://broadinstitute. github.io/picard/), while gene variants were identified by base recalibration variant calling with Haplotype Caller, variant quality score recalibration, and variant annotation using ANNOVAR software. *RAD21L1* mutations were determined by the following four criteria: a frequency less than 5% in three public databases, including 1000 genomes variant database, NHLBI‐GO exome sequencing project and Exome Aggregation Consortium; deleterious variants by prediction; homozygous variants with priority; phenotype relevancy with male infertility. Homozygosity mapper was drawn with homozygosity mapping, and homozygous variants at homozygous regions >5.0 Mb were regarded as priority.

### Statistical Analysis

Statistical analyses and risk analysis of *RAD21L1* mutations were completed using GraphPad Prism 8.0 and SPSS 20.0, respectively. All results were presented as the mean ± SD from three or more independent experiments. Statistically significant differences between two groups were conducted using Student's *t*‐test, and risk analysis was conducted utilizing chi‐square test and logistic regression. The *p*< 0.05 was chosen as statistical significance.

## Conflict of Interest

The authors declare no conflict of interest.

## Author Contributions

C.H. completed experiments and wrote the manuscript. Y.C., W.C., and C.L. assisted with experiments. Z.H. was in charge of experiments, revising the manuscript and data analysis. All authors read the article and agreed to its publication.

## Supporting information



Supporting Information

## Data Availability

The data that support the findings of this study are available from the corresponding author upon reasonable request.
