# Supplementary material for: RAD21L1 Is Sufficient and Effective for Reprogramming Human Sertoli Cells to Phenotypic Spermatogonial Stem Cells Through DNA Methylation and Essential for Male Fertility
Source: Adv Sci (Weinh). 2025 Oct 16;12(45):e17491. doi: 10.1002/advs.202417491 (PMC12677610; doi:10.1002/advs.202417491)
Supplement: Supplementary file 1 — Supporting Information [file ADVS-12-e17491-s001.pdf]

Supplemental Information for advs.202417491R3

RAD21L1 Is Sufficient and Effective for Directly Reprogramming Sertoli Cells to Human Spermatogonial Stem Cells through DNA Methylation and *RAD21L1* Mutations Are Associated with Male Infertility

Caimei He, Yinghong Cui, Wei Chen, Chunyun Li, and Zuping He

Table S1-S6

Figures S1-S5

Table S1-S6

Table S1. *RAD21L1* variants identified in NOA patients

**Allele frequency in human population/Functional prediction**						
**gnomAD_exome**	**ExAC**	**ExAC_nontcga**	**1000genomes**	**ESP6500**	**Kaviar**	**CG69**
0.0309	0.0253	0.0228	0.0169728	0.0397	0.010084	0.007
5.79E-05	4.80E-05	6.28E-05	0.00019968	NA	1.29E-05	NA
0.0001	5.06E-05	6.66E-05	0.000599042	NA	2.59E-05	NA
1.32E-05	NA	NA	NA	NA	NA	NA
0.0001	5.06E-05	6.66E-05	0.000599042	NA	2.59E-05	NA
2.82E-05	NA	NA	NA	NA	6.50E-06	NA
0.0001	3.23E-05	5.06E-05	0.000599042	NA	2.59E-05	NA
VEST3	Tolerable (0.081)	Tolerable (0.176)	Tolerable (0.3)		Tolerable (0.11)	
Eigen	Tolerable (-0.604)	Tolerable (-0.79)	Tolerable (-0.322)		Tolerable (-1.128)	
Mutation Assessors	Low (1.245)	Low (1.59)	Low (0.94)		Low (1.42)	
GenoCanyou	Tolerable(0.004)	Damaging (1)	Tolerable(0.884)		Tolerable(0)	
FitCons	Tolerable(0.554)	Tolerable(0.495)	Tolerable(0.497)		Tolerable(0.487)	
REVEL	Tolerable(0.172)	Tolerable(0.042)	NA		Tolerable(0.048)	
Mutation Taster	Polymorphism(1)	Polymorphism(1)	Tolerable		Polymorphism(0.995)	
CADD	Tolerable(8.531)	Tolerable(9.391)	Tolerable(18.87)		Tolerable(4.815)	
Polypehn-2-HDIY	Possibly_damaging (0.598)	Benign(0.002)	Benign(0.001)		Benign(0)	
SIFT	Tolerable(0.177)	Tolerable(0.382)	Tolerable(0.286)		Tolerable(0.1)	

NCBI Reference Sequences (RefSeq) number of *RAD21L1* was: NM_001136566.3

NA: Not available

Table S2. The sequences of *DAZL*, *DAZ2*, *BOULE* and *RAD21L1* genes

The sequence of *DAZL* gene (12,552 bp)	ATGGCGGCTCCCTCGTGTGGCGGCGACAGAAAAGCTCGCCTGA<br>CGCCATCTTTGCCGCACGAGTCTACTGCAAATCCTGAAACTCC<br>AAACTCAACCATCTCCAGAGAGGCCAGCACCCAGTCCTCATCA<br>GCTGCAACCAGCCAAGGCTATATTTTACCAGAAGGCAAAATCA<br>TGCCAAACACTGTTTTTGTTGGAGGAATTGATGTTAGGATGGA<br>TGAAACTGAGATTAGAAGCTTCTTTGCTAGATATGGTTCAGTG<br>AAAGAAGTGAAGATAATCACTGATCGAACTGGTGTGTCCAAA<br>GGCTATGGATTTGTTTCATTTTTTAATGACGTGGATGTGCAGAA<br>GATAGTAGAATCACAGATAAATTTCCATGGTAAAAAGCTGAAG<br>CTGGGCCCTGCAATCAGGAAACAAAATTTATGTGCTTATCATG<br>TGCAGCCACGTCCTTTGGTTTTTAATCATCCTCCTCCACCACAG<br>TTTCAGAATGTCTGGACTAATCCAAACACTGAAACTTATATGC<br>AGCCCACAACCACGATGAATCCTATAACTCAGTATGTTCAGGC<br>ATATCCTACTTACCCAAATTCACCAGTTCAGGTCATCACTGGAT<br>ATCAGTTGCCTGTATATAATTATCAGATGCCACCACAGTGGCC<br>TGTTGGGGAGCAAAGGAGCTATGTTGTACCTCCGGCTTATTCA<br>GCTGTTAACTACCACTGTAATGAAGTTGATCCAGGAGCTGAAG<br>TTGTGCCAAATGAATGTTCAGTTCATGAAGCTACTCCACCCTCT<br>GGAAATGGCCCACAAAAGAAATCTGTGGACCGAAGCATACAA<br>ACGGTGGTATCTTGTCTGTTTAATCCAGAGAACAGACTGAGAA<br>ACTCTGTTGTTACTCAAGATGACTACTTCAAGGATAAAAGAGT<br>GCATCACTTTAGAAGAAGTCGGGCAATGCTTAAATCTGTT
The sequence of *DAZ2* gene (13,281 bp)	ATGTCTGCTGCAAATCCTGAGACTCCAAACTCAACCATCTCCA<br>GAGAGGCCAGCACCCAGTCTTCATCAGCTGCAGCTAGCCAAGG<br>CTGGGTGTTACCAGAAGGCAAAATCGTGCCAAACACTGTTTTT<br>GTTGGTGGAATTGATGCTAGGATGGATGAAACTGAGATTGGAA<br>GCTGCTTTGGTAGATACGGTTCAGTGAAAGAAGTGAAGATAAT<br>CACGAATCGAACTGGTGTGTCCAAAGGCTATGGATTTGTTTCG<br>TTTGTTAATGACGTGGATGTCCAGAAGATAGTAGGATCACAGA<br>TACATTTCCATGGTAAAAAGCTGAAGCTGGGCCCTGCAATCAG<br>GAAACAAAAGTTATGTGCTCGTCATGTGCAGCCACGTCCTTTG<br>GTAGTTAATCCTCCTCCTCCACCACAGTTTCAGAACGTCTGGCG<br>GAATCCAAACACTGAAACCTACCTGCAGCCCCAAATCACGCCG<br>AATCCTGTAACTCAGCACGTTCAGGCTTATTCTGCTTATCCACA<br>TTCACCAGGTCAGGTCATCACTGGATGTCAGTTGCTTGTATATA<br>ATTATCAGGAATATCCTACTTATCCCGATTCAGCATTTCAGGTC<br>ACCACTGGATATCAGTTGCCTGTATATAATTATCAGCCATTTCC<br>TGCTTATCCAAGATCACCATTTCAGGTCACTGCTGGATATCAGT<br>TGCCTGTATATAATTATCAGGCATTTCCTGCTTATCCAAATTCA<br>CCATTTCAAGTCGCCACTGGATATCAGTTCCCTGTATACAATTA<br>TCAGCCATTTCCTGCTTATCCAAGTTCACCATTTCAGGTCACTG<br>CTGGATATCAGTTGCCTGTATATAATTATCAGGCATTTCCTGCT

	TATCCAAATTCACCATTTCAAGTCGCCACTGGATATCAGTTCCC<br>TGTATACAATTATCAGGCATTTCCTGCTTATCCAAATTCACCAG<br>TTCAGGTCACCACTGGATATCAGTTGCCTGTATACAATTATCAG<br>GCATTTCCTGCTTATCCAAATTCACCAGTTCAGGTCACCACTGG<br>ATATCAGTTGCCTGTATACAATTATCAGGCATTTCCTGCTTATC<br>CAAGTTCACCATTTCAGGTCACCACTGGATATCAGTTGCCTGTA<br>TATAATTATCAGGCATTTCCTGCTTATCCAAGTTCACCATTTCA<br>GGTCACCACTGGATATCAGTTGCCTGTATATAATTATCAGGCA<br>TTTCCTGCTTATCCAAGTTCACCATTTCAGGTCACCACTGGATA<br>TCAGTTGCCTGTATATAATTATCAGGCATTTCCTGCTTATCCAA<br>GTTCACCATTTCAGGTCACCACTGGATATCAGTTGCCTGTATAT<br>AATTATCAGGCATTTCCTGCTTATCCAAGTTCACCATTTCAGGT<br>CACCACTGGATATCAGTTGCCTGTATATAATTATCAGGCATTTC<br>CTGCTTATCCAAGTTCACCATTTCAGGTCACCACTGGATATCAG<br>TTGCCTGTATATAATTATCAGGCATTTCCTGCTTATCCAAATTC<br>AGCAGTTCAGGTCACCACTGGATATCAGTTCCATGTATACAAT<br>TACCAGATGCCACCGCAGTGCCCTGTTGGGGAGCAAAGGAGA<br>AATCTGTGGACCGAAGCATACAAATGGTGGTATCTTGTCTGTT<br>TAATCCAGAGAAGAGAC
The sequence of the *BOULE* gene (12,624 bp)	ATGCAAACATCAAACCAGATGCAAACAGATTCATTATCTCCAT<br>CCCCTAATCCTGTGTCACCTGTGCCTTTGAATAACCCAACAAGT<br>GCCCCAAGATATGGAACAGTGATCCCTAATCGCATCTTTGTAG<br>GAGGAATTGATTTTAAGACAAACGAAAGTGATTTAAGAAAATT<br>TTTTTCCCAGTATGGGTCTGTGAAAGAAGTGAAGATTGTAAAT<br>GACAGAGCTGGAGTATCCAAAGGGTATGGTTTCGTCACTTTTG<br>AAACACAAGAAGATGCACAAAAAATTTTACAAGAGGCTGAAA<br>AACTTAATTATAAGGATAAGAAGCTGAACATTGGTCCAGCAAT<br>AAGAAAACAACAAGTAGGGATCCCTCGTTCTAGTATAATGCCA<br>GCAGCTGGAACAATGTATCTAACAACTTCAACTGGATATCCTT<br>ATACTTACCATAATGGTGTTGCTTATTTTCATACTCCAGAGGTA<br>ACTTCGGTCCCACCGCCTTGGCCTTCACGTTCTGTATGTAGCTC<br>CCCTGTGATGGTAGCTCAGCCCATTTATCAGCAACCTGCATATC<br>ACTACCAGGGAATTAAACAATGTCATACAAGAAGATGGATGG<br>ACTCTTTGCTTCCTTTTACCAAATGTGATGAGGCCACCACACAG<br>TATTTACCAGGACAGTGGCAGTGGAGTGTTCCTCAGCCTTCTG<br>CCTCTTCTGCTCCATTCTTATACCTGCAACCTTCTGAGGTTATTT<br>ATCAACCAGTGGAAATTGCACAGGATGGTGGATGTGTTCCTCC<br>TCCACTGTCTCTGATGGAAACTTCAGTTCCAGAGCCTTATTCTG<br>ATCATGGAGTTCAAGCAACATATCACCAGGTTTATGCTCCAAG<br>TGCCATCACTATGCCTGCGCCTGTGATGCAGCCTGAGCCAATT<br>AAATGTGGAGCATTCATTATTAAGACAATTGGGCAGCTCTATT<br>CCAGCTCAACTGATTTCTTGTCCAATGATCCTTGCGTGGCCGAG<br>ATCCAGCTTCAACGAACCAGC
The sequence of	ATGTTCTACACACATGTGCTTATGAGTAAACGAGGGCCATTGG

RAD21L1 gene (11,976 bp)	CCAAAATATGGCTTGCAGCTCACTGGGAGAAGAAACTCACAA<br>AGGCCCATGTATTTGAATGTAATCTAGAGATAACCATTGAAAA<br>AATTCTTTCACCCAAGGTGAAAATAGCACTTCGAACTTCAGGA<br>CACCTTCTTTTGGGAGTTGTTCGAATCTATAACAGGAAGGCAA<br>AATATCTTTTGGCAGATTGCAGTGAAGCATTTCTTAAAATGAA<br>GATGACATTTTGCCCAGGACTGGTTGACCTTCCAAAAGAGAAT<br>TTTGAAGCATCTTACAATGCTATCACATTGCCAGAAGAATTTC<br>ATGATTTTGACACCCAAAATATGAATGCTATTGATGTTTCAGA<br>ACACTTTACTCAGAACCAAAGCAGACCAGAAGAAATCACTCTT<br>AGAGAAAATTTTGACAATGATCTAATTTTCCAAGCTGAGAGCT<br>TTGGGGAGGAATCTGAAATTCTCAGAAGACATAGCTTCTTTGA<br>TGACAACATATTACTGAATTCCAGTGGTCCTTTAATTGAACATA<br>GTTCTGGAAGCCTCACTGGAGAACGATCTCTATTCTATGACAG<br>TGGAGATGGGTTTGGAGATGAAGGAGCTGCAGGAGAAATGAT<br>TGACAATCTATTGCAAGATGATCAGAATATCCTGTTAGAAGAC<br>ATGCATTTGAACAGAGAAATTTCCCTGCCTTCTGAGCCTCCCA<br>ATAGTTTAGCAGTTGAACCAGATAACTCAGAGTGTATATGTGT<br>ACCTGAAAATGAAAAAATGAATGAAACAATATTATTATCAACT<br>GAAGAGGAAGGATTTACCCTTGATCCAATTGATATTTCAGACA<br>TTGCTGAGAAAAGGAAAGGCAAAAAGAGGAGATTGCTCATAG<br>ATCCTATCAAGGAGCTCAGTAGCAAAGTTATACATAAACAGCT<br>TACTTCCTTTGCGGACACACTCATGGTTTTGGAACTTGCACCTC<br>CTACCCAAAGATTGATGATGTGGAAGAAGAGGGGAGGAGTGC<br>ATACACTTCTGTCAACTGCTGCCCAGGATTTGATTCATGCTGAA<br>CTGAAAATGTTGTTTACAAAATGCTTTCTGTCCTCTGGCTTTAA<br>ACTTGGAAGAAAAATGATACAGAAGGAGTCAGTAAGGGAAGA<br>AGTGGGAAACCAAAATATAGTAGAGACATCCATGATGCAAGA<br>GCCAAATTACCAGCAAGAGTTAAGTAAACCCCAAACTTGGAA<br>GGATGTGATTGGTGGATCTCAGCATAGCTCTCATGAGGATACC<br>AATAAAAATATTAACTCTGAGCAGGATATTGTTGAAATGGTGT<br>CTTTAGCTGCTGAGGAATCATCTTTAATGAATGACTTATTTGCA<br>CAAGAAATTGAATATAGTCCAGTTGAATTGGAGTCATTGAGCA<br>ATGAAGAAAATATTGAGACAGAGAGATGGAATGGAAGAATAC<br>TTCAGATGTTAAATCGTTTACGGGAATCTAACAAGATGGGAAT<br>GCAGTCCTTTAGTCTGATGAAGCTCTGTAGAAATAGTGACCGA<br>AAACAAGCAGCTGCCAAATTTTATAGCTTTCTTGTCCTAAAGA<br>AACAGCTGGCTATTGAGCTGAGCCAGAGTGCTCCCTATGCAGA<br>TATTATAGCTACGATGGGACCAATGTTTTATAACATA

Table S3. Antibodies, tissue samples, primary cells, cell line, and siRNAs used in this study

Regents	Sources	Catalogue numbers
**Antibodies**		
SCF	Abcam	Cat#ab2603
WT1	CST	Cat#83535
SOX9	GeneTex	Cat#GTX01545
GATA4	Abcam	Cat#ab307823
AR	Abcam	Cat#ab198394
BMP4	Abcam	Cat#ab124715
GDNF	Abcam	Cat#ab176564
ACTB	CST	Cat#4967
DAZL	Abcam	Cat#ab34139
DAZ2	CST	Cat#Ab97772
BOULE	LSBio	Cat#LS-B7334
UCHL1	Bio-Rad	Cat#MCA4750
UCHL1	CST	Cat#13179S
GPR125	Abcam	Cat#Ab51705
GFRA1	R&D	Cat#260714
CD90	BioSciences	Cat#LS-C45431
MAGEA4	CST	Cat#82491
SV40	Santa Cruz bioecology	Cat#B0703
HumNuc	Abcam	Cat#ab191181
PCNA	CST	Cat#13110s
PCNA	Abcam	Cat#ab29
PLZF	Abcam	Cat#ab104854
SYCP3	Abcam	Cat#Ab15093
MHL1	BD pharming	Cat#51-1327GR
FLAG	Proteintech	Cat#66008-3-Ig
RAD21L1	Proteintech	Cat#24206-1-AP
RAD21L1	Bioss	Cat#bs-21139R
DNMT1	Thermo Scientific	Cat#PA5-30581
PE anti-human CD90	BioLegend	Cat#328109
Goat Anti-Rabbit HRP	Beyotime/Biotechnology	Cat#A0208
GoatAnti-Mouse HRP	Beyotime/Biotechnology	Cat#A0126
Alexa Fluor 488	Thermo scientific	Cat#A21202
Alexa Fluor 488	Thermo scientific	Cat#A21206
Alexa Fluor 555	Thermo scientific	Cat#A31570
Alexa Fluor 555	Thermo scientific	Cat#A31572
IgG	CST	Cat#2729S
IgG	Proteintech	Cat#B900620
**Biological samples**		

Testicular tissues	N/A	From OA and NOA patients
Peripheral blood	N/A	From NOA patients
**Chemicals, peptides, and recombinant proteins**		
FBS	Gibco	Lot.1640960
DMEM/F-12	Gibco	Cat#21331020
Penicillin-Streptomycin	Sigma-Aldrich	Cat# P4333
Trypsin	Gibco	Cat# 25200114
Hyaluronidase	Sigma-Aldrich	Cat# H1115000
BSA	Sigma-Aldrich	Cat# 10711454001
Protein Ladder	Thermos scientific	Cat#26616
RA	Proteintech	Cat#302-79-4
SCF	Merch	Cat#300-07-250UG
Busulfan	Sigma-Aldrich	Cat# 55-98-1
16% formaldehyde	Cell Signaling Technology	Cat# 12606S
RIPA	Beyotime	Cat# P0013B
PMSF	Beyotime	Cat# ST506
RNAiso Plus regent	Takara	Cat# 9109
DNase I	Gibco	Cat# 18047019
CollagenaseType IV	Gibco	Cat# 17104019
HiScript II Q RT SuperMix	Vazyme	Cat# R223-01
Power SYBR Green PCR Master Mix	Applied Biosystems	Cat# 4367659
lipofectamine 3000	Life Technologies	Cat# L3000150
**Critical commercial assays**		
Y chromosome microdeletion test kit	Tellgen	Cat# SLAN-96S/48P
**Experimental models: Cell line and primary cells**		
Cells: Human primary Sertoli cells	N/A	From OA and NOA patients
Cell line: Human SSC line	Hou, 2015	N/A
**Oligonucleotides**		
RAD21L1 siRNAs	This paper	N/A
Primers	This paper	N/A
**Software and algorithms**		
BD FACSDiva software	BD Biosciences	N/A
FlowJo v10	BD Biosciences	https://www.flowjo.com
Prism 8	GraphPad	https://www.graphpad.com

Table S4. The primer sequences of genes for qPCR and RT-PCR

Genes		Primer sequences (5'-3')	Product sizes (bp)
GAPDH	Forward	AATCCCATCACCATCTTCC	382
	Reverse	CATCACGCCACAGTTTCC	
GAPDH	Forward	GTCTCCTCTGACTTCAACAGCG	131
	Reverse	ACCACCCTGTTGCTGTAGCCAA	
AR	Forward	AATGAAGATGAGTCACCAGAGG	227
	Reverse	CAAAGGAAGATTGGTGGTTAGC	
BMP4	Forward	TTTGTTCAAGATTGGCTGTC	324
	Reverse	AGATCCCGCATGTAGTCC	
SOX9	Forward	AGGAAGCTCGCGGACCAGTAC	148
	Reverse	GGTGGTCCTTCTTGTGCTGCAC	
SCF	Forward	AATCCTCTCGTCAAAACTGAAGG	163
	Reverse	CCATCTCGCTTATCCAACAATGA	
GATA1	Forward	CACGACACTGTGGCGGAGAAAT	140
	Reverse	TTCCAGATGCCTTGCGGTTTCG	
FSHR	Forward	TCTGCTGGTTCTGTTTCA	182
	Reverse	CATTCCTTGGATGGGTGT	
GDNF	Forward	CGCCGAAGACCGCTCCCTCG	103
	Reverse	ATCCATGACATCATCGAACTGAT	
WT1	Forward	CGAGAGCGATAACCACACAACG	138
	Reverse	GTCTCAGATGCCGACCGTACAA	
DAZL	Forward	TGACGTGGATGTGCAGAAGATAG	123
	Reverse	ACCAAAGGACGTGGCTGCACAT	
DAZ2	Forward	CAGAACGTCTGGCGGAATCCAA	130
	Reverse	GACATCCAGTGATGACCTGACC	
BOULE	Forward	ACTCCAGAGGTAACTTCGGTCC	156
	Reverse	CTGAGGAACACTCCACTGCCAC	
GFRA1	Forward	CCAAAGGGAACAACTGCCTG	121
	Reverse	CGGTTGCAGACATCGTTGGA	
GPR125	Forward	GCGTCATTACGGTCTTTGGAA	199
	Reverse	ACGGCAATTCAAGCGGAGG	
UCHL1	Forward	AGCTGAAGGGACAAGAAGTTAG	265
	Reverse	TTGTCATCTACCCGACATTGG	
PLZF	Forward	CGGTTCCTGGATAGTTTGC	317
	Reverse	GGGTGGTCGCCTGTATGT	
MAGEA4	Forward	CTTACCCACTACCATCAGCTTC	212
	Reverse	TGATGACTCTCTCCAGCATTTC	
RET	Forward	CTCGTTCATCGGGACTTG	126
	Reverse	ACCCTGGCTCCTCTTCAC	
THY1	Forward	CAGAAGGTGACCAGCCTAAC	233
	Reverse	TTGCTAGTGAAGGCGGATAAG	

TEX15	Forward	GACTCTCAGGAACAACAGCAAGA	141
	Reverse	TTCGCTTTTGGGATGAGATCCTG	
CFAP299	Forward	GCAAGACTGGCTGAAAGAGCTC	119
	Reverse	CTCAGTTTTCCACTGCGATTGTC	
RAD21L1	Forward	GGAGTCAGTAAGGGAAGAAGTGG	134
	Reverse	GAGAGCTATGCTGAGATCCACC	
SLC45A2	Forward	CTTTGCATCAGCCACCTCATTGG	153
	Reverse	TCCAACCTCGACTCCTCTTTCG	
DACH2	Forward	GCCAGGACTTATCACTCCGACA	138
	Reverse	TCATCAGGCTCTGATTCGGTCC	
HOXB9	Forward	TGCGAAGGAAGCGAGGACAAAG	128
	Reverse	TCCTTCTCTAGCTCCAGCGTCT	
ESRRB	Forward	CCGCACACGAGGCACAGG	105
	Reverse	CCGCAAGAGCTACGAGGACTG	
OCT4	Forward	CCTGAAGCAGAAGAGGATCACC	331
	Reverse	AAAGCGGCAGATGGTCGTTTGG	
LIN28A	Forward	CCAGTGGATGTCTTTGTGCACC	125
	Reverse	GTGACACGGATGGATTCCAGAC	
DLX5	Forward	TACCCAGCCAAAGCTTATGCCG	138
	Reverse	GCCATTCACCATTCTCACCTCG	
WNT9	Forward	AGTGCCAGTTCCAGTTCCGCTT	114
	Reverse	AGGAGATGGCATAGAGGAAGGC	
FGF2	Forward	AGCGGCTGTACTGCAAAAACGG	139
	Reverse	CCTTTGATAGACACAACTCCTCT	
PRDM16	Forward	CAGCCAATCTCACCAGACACCT	146
	Reverse	GTGGCACTTGAAAGGCTTCTCC	
PRDM1	Forward	CAGTTCCTAAGAACGCCAACAGG	123
	Reverse	GTGCTGGATTCACATAGCGCATC	
EZH2	Forward	GACCTCTGTCTTACTTGTGGAGC	115
	Reverse	CGTCAGATGGTGCCAGCAATAG	
DNMT1	Forward	AGGTGGAGAGTTATGACGAGGC	146
	Reverse	GGTAGAATGCCTGATGGTCTGC	
DOT1L	Forward	GTTCCTGGCATACACAAAGACCC	155
	Reverse	GCTGAAACAGCCTCCTGATCTC	
CREST	Forward	CGCAGCAGCAGACGTACTC	204
	Reverse	GGCCCTGTTCATAGCCGTAG	
SYCP3	Forward	TGCAGAAAGCTGAGGAACAA	247
	Reverse	TGCTGCTGAGTTTCCATCAT	
PIWIL2	Forward	CTTTCCGACCATCGTTCA	431
	Reverse	TCTTCCAAGCGTCCTACT	
MHL1	Forward	TGAGGAAGGGAACCTGATTG	245
	Reverse	TCCAGGAGTTTGGAATGGAG	
rH2AX	Forward	ACCTCACCGCTGAGATCCT	167
	Reverse	CTGGATGTTGGGCAGGAC	

Table S5. The primer sequences of genes for Multiplex PCR

Genes	Primer sequences (5'-3')	
sY84	Forward	5'-biotin AGACAAAACTTTGAAAGGAG-3'
	Reverse	5'-AAGGGATTTTACATACAGACATA-3'
sY86	Forward	5'-biotin-AGACTATGCTTCAGCAGGTC-3'
	Reverse	5'-CAGTCTTTGGGATTTCTTT-3'
sY127	Forward	5'-biotin-GGCTCACAAACGAAAAGAAA-3'
	Reverse	5'-CTGCAGGCAGTAATAAGGGA-3'
sY134	Forward	5'-biotin-GTCTGCCTCACCATAAAACG-3'
	Reverse	5'-ACCACTGCCAAAACTTTCAA-3'
sY254	Forward	5'-biotin-GGGTGTTACCAGAAGGCAAA-3'
	Reverse	5'-GAACCGTATCTACCAAAGCAGC-3'
sY255	Forward	5'-biotin GTTACAGGATTCGGCGTGAT-3'
	Reverse	5'-CTCGTCATGTGCAGCCAC-3'
SRY	Forward	5'-biotin-GAATATTCCCGCTCTCCGGA-3'
	Reverse	5'-GCTGGTGCTCCATTCTTGAG-3'
ZFX/ZFY	Forward	5'-biotin-CTGACCAGCAAGGCAGAGAA-3'
	Reverse	5'-GAGAATATGCGACTTAGAAC-3'

Table S6. The sequences of *RAD21L1* siRNA oligonucleotides

siRNAs	Sequences (5'-3')	
RAD21L1 siRNA1	sense	GCUCAGUAGCAAAGUUAUA
	antisense	UAUAACUUUGCUACUGAGC
RAD21L1 siRNA2	sense	CCAGCAAGAGUUAAGUAAA
	antisense	UUUACUUAACUCUUGCUGG
RAD21L1 siRNA3	sense	GCUGAGGAAUCAUCUUUAA
	antisense	UUAAAGAUGAUUCCUCAGC
Control siRNA	sense	UUCUCCGAACGUGUCACGU
	antisense	ACGUGACACGUUCGGAGAA
FAM-siRNA	sense	UUCUCCGAACGUGUCACGU
	antisense	ACGUGACACGUUCGGAGAA

Figures S1-S5

Figure S1

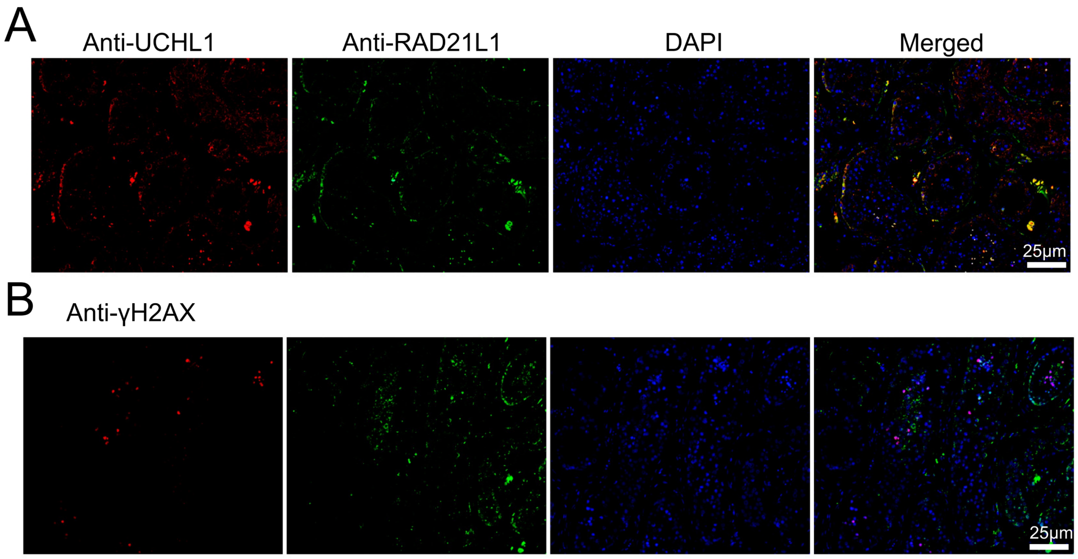

Figure S1. The colocalization of RAD21L1 with UCHL1 and γH2AX in human testis tissues. (A) Representative images of double immunostaining illustrated the co-expression of RAD21L1 and UCHL1 in human SSCs of human testicular tissues. (B) Representative images of double immunostaining indicated RAD21L1 and γH2AX co-expression in spermatocytes of human testicular tissues. Scale bars in (A) and (B): 25 μm.

Figure S2

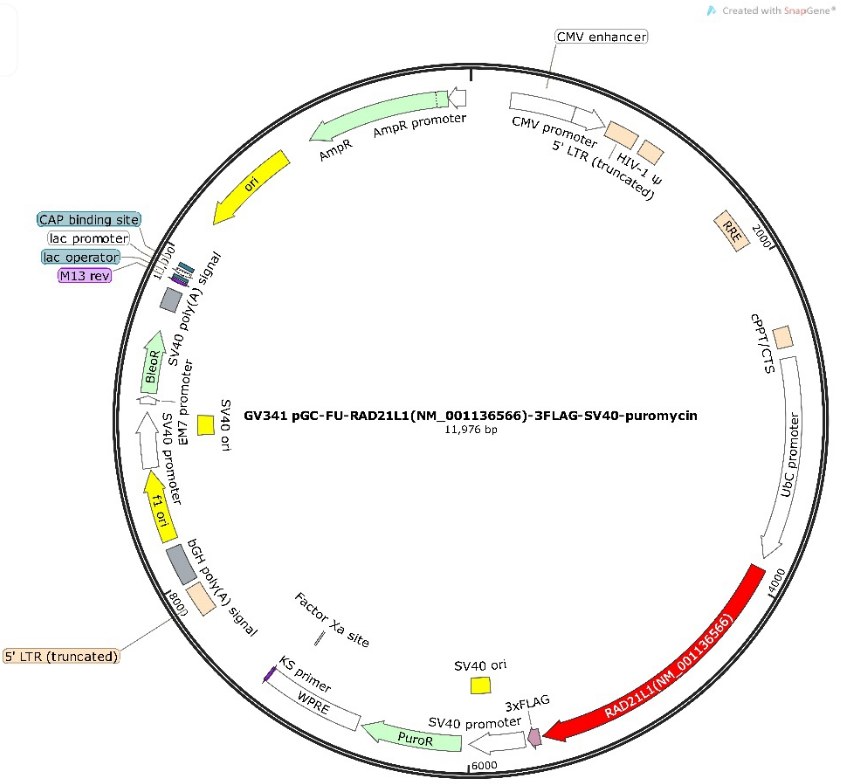

Figure S2. The map showed the structure of lentivirus overexpressing *RAD21L1* plasmid. (A) The plasmids overexpressing *RAD21L1* gene was fused with a flag at Shanghai GeneChem Co. Ltd. The promoter of *RAD21L1* gene was driven by CMV promoter and puromycin-resistant. The size of *RAD21L1* gene was 11,976 bp, and it was carried with the flag tag.

Figure S3

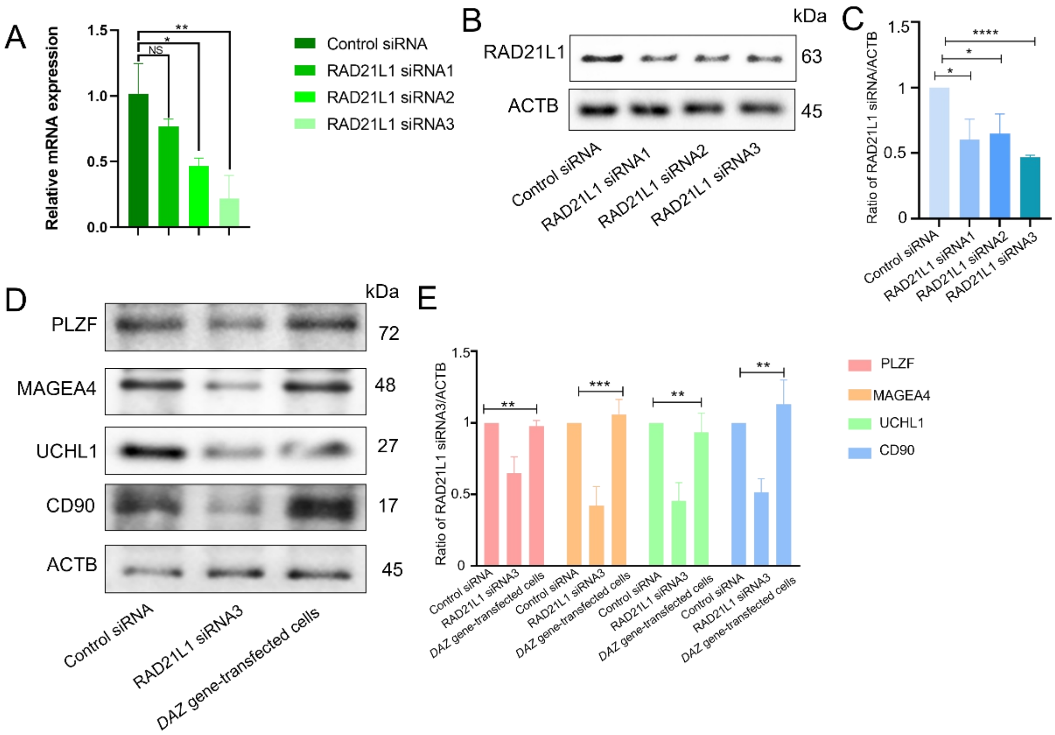

Figure S3. RAD21L1 knockdown inhibited the reprogramming human Sertoli cells into SSCs by overexpressing *DAZ* family genes. (A) The transfection efficiency of *RAD21L1*-siRNAs was measured by qPCR displayed the level of *RAD21L1* mRNA in the *DAZ* genes-transfected cells with the treatment of control siRNA and *RAD21L1*-siRNA1-3. (B-C) Western blots showed the changes in the RAD21L1 protein in the *DAZ* genes-transfected cells with the treatment of control siRNA and *RAD21L1*-siRNA 1-3. (D-E) Western blots exhibited the level of changes in PLZF, MAGEA4, UCHL1 and CD90 proteins in the *DAZ* genes-transfected cells at 72 hours after transfection of control siRNA and *RAD21L1* siRNA3.

Figure S4

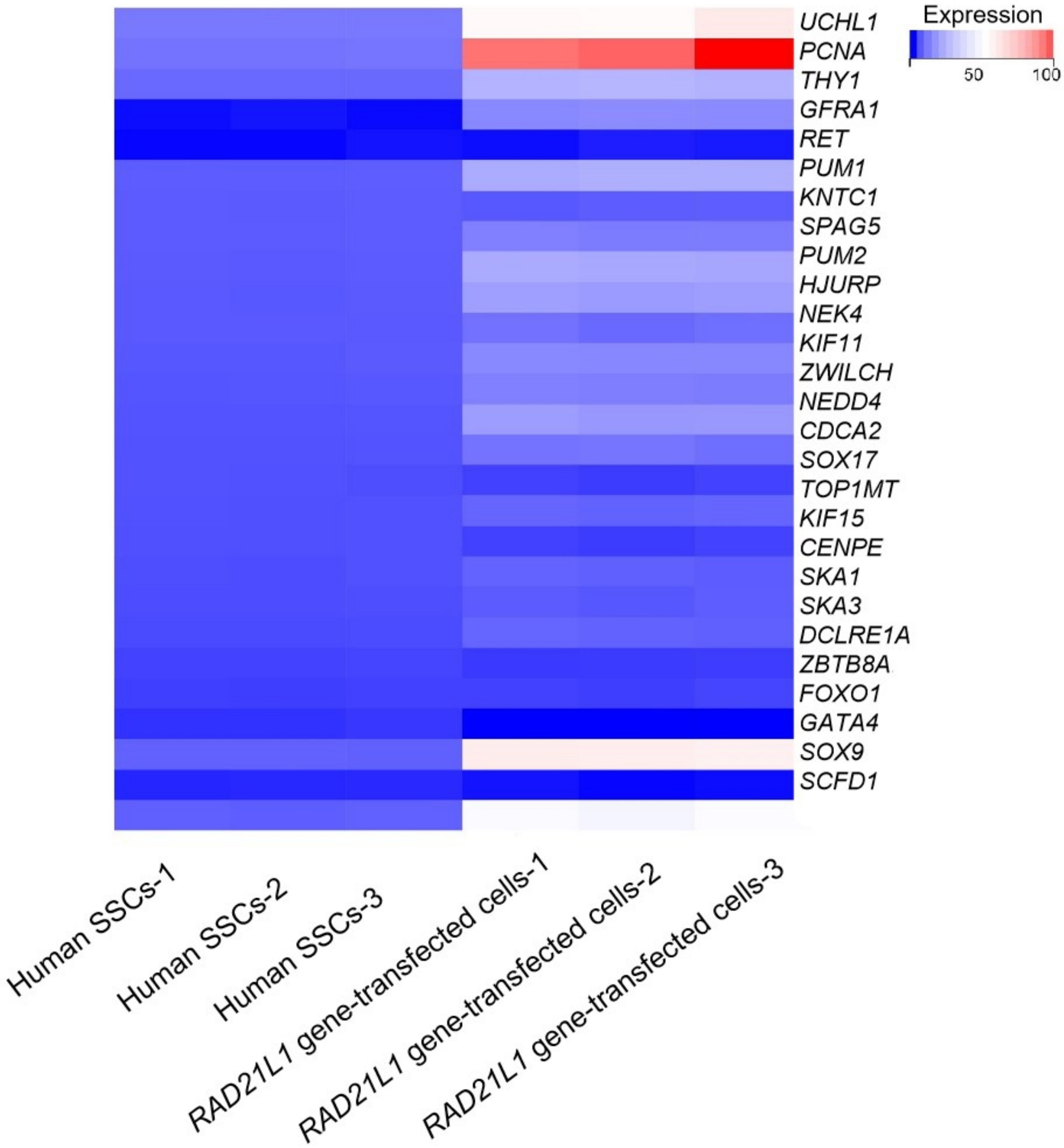

Figure S4. *RAD21L1* gene-transfected cells had similar transcriptomes to human SSCs. Our heatmap analysis showed that no significant difference was observed in transcripts of *UCHL1*, *THY1*, *GFRA1*, and *PCNA* between *RAD21L1* gene-transfected cells and human SSC line.

Figure S5

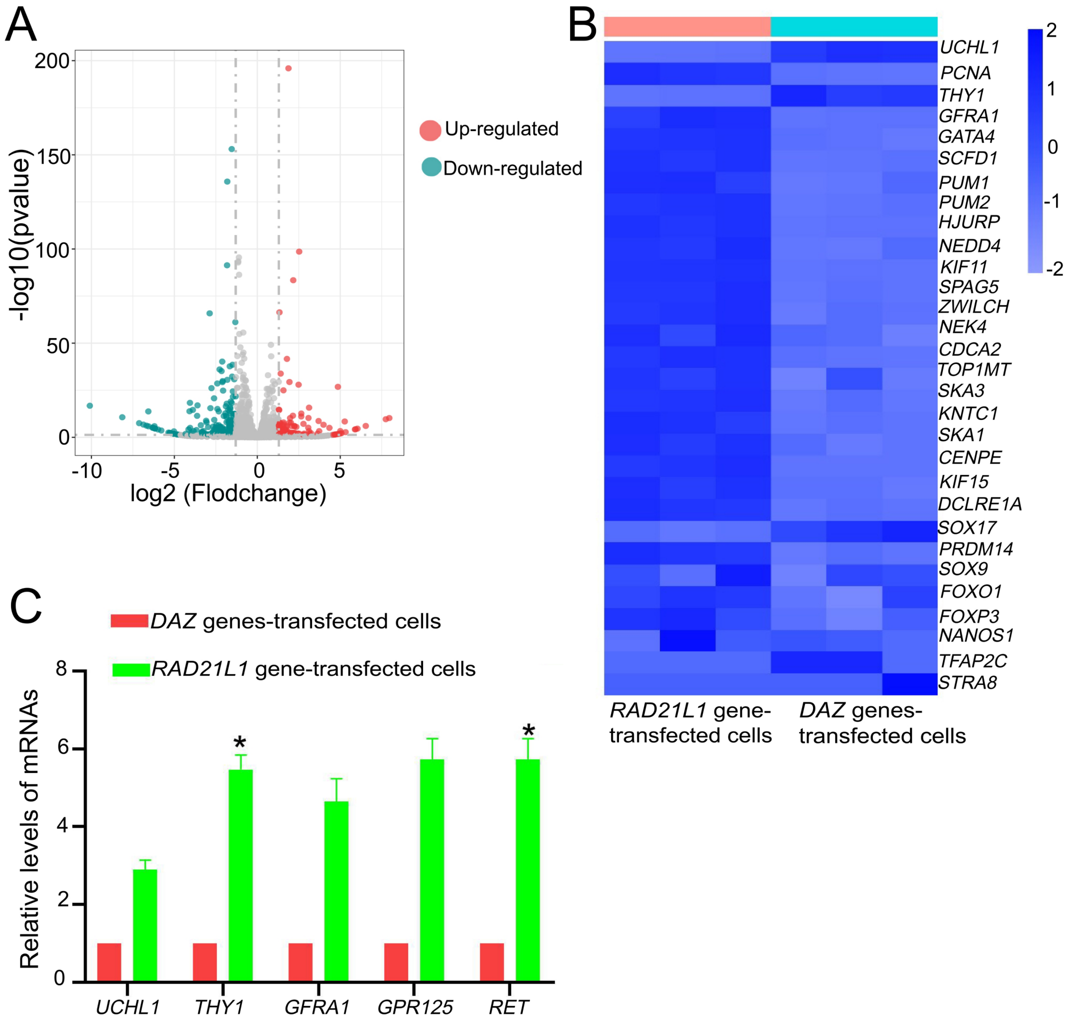

Figure S5. *RAD21L1* gene-transfected cells possessed similar transcriptomes to *DAZ* genes-transfected cells derived from human Sertoli cells. (A)Volcano plot demonstrated the DEGs between *RAD21L1* gene-transfected cells and *DAZ* genes-transfected cells derived from human Sertoli cells. (B) Our heatmap analysis showed higher transcription levels of *UCHL1* and *THY1* mRNA in *RAD21L1* gene-transfected cells than *DAZ* genes-transfected cells. (C) The qPCR results indicated that mRNA expression levels of *UCHL1* and *THY1* were significantly

elevated in *RAD21L1* gene-transfected cells from human Sertoli cells compared with *DAZ* genes-transfected cells.
